# Increased intestinal permeability exacerbates sepsis through reduced hepatic SCD-1 activity and dysregulated iron recycling

**DOI:** 10.1038/s41467-019-14182-2

**Published:** 2020-01-24

**Authors:** Manish Kumar, Aralia Leon Coria, Steve Cornick, Björn Petri, Shyamchand Mayengbam, Humberto B. Jijon, France Moreau, Jane Shearer, Kris Chadee

**Affiliations:** 1 Department of Microbiology, Immunology and Infectious Diseases, Snyder Institute for Chronic Diseases, University of Calgary Health Sciences Centre, 3330 Hospital Drive NW, Calgary, AB T2N4N1 Canada; 2 Snyder Institute for Chronic Diseases, Mouse Phenomics Resource Laboratory, University of Calgary, Calgary, AB T2N4N1 Canada; 3 Faculty of Kinesiology, University of Calgary, 2500 University Drive, NW Calgary, AB T2N1N4 Canada; 4 Inflammatory Bowel Disease Unit, Division of Gastroenterology and Hepatology, Cumming School of Medicine, University of Calgary, Calgary, AB Canada; 5 Department of Biochemistry and Molecular Biology, Cumming School of Medicine, University of Calgary, 3330 Hospital Drive NW, Calgary, AB T2N 4N1 Canada

**Keywords:** Infection, Chronic inflammation, Mucosal immunology, Microbial communities

## Abstract

Inflammatory bowel disease is associated with changes in the mucosal barrier, increased intestinal permeability, and increased risk of infections and sepsis, but the underlying mechanisms are incompletely understood. Here, we show how continuous translocation of gut microbial components affects iron homeostasis and facilitates susceptibility to inflammation-associated sepsis. A sub-lethal dose of lipopolysaccharide results in higher mortality in Mucin 2 deficient (*Muc2*^−/−^) mice, and is associated with elevated circulatory iron load and increased bacterial translocation. Translocation of gut microbial components attenuates hepatic stearoyl CoA desaturase-1 activity, a key enzyme in hepatic de novo lipogenesis. The resulting reduction of hepatic saturated and unsaturated fatty acid levels compromises plasma membrane fluidity of red blood cells, thereby significantly reducing their life span. Inflammation in *Muc2*^−/−^ mice alters erythrophagocytosis efficiency of splenic macrophages, resulting in an iron-rich milieu that promotes bacterial growth. Our study thus shows that increased intestinal permeability triggers a cascade of events resulting in increased bacterial growth and risk of sepsis.

## Abstract (web summary)

Here Kumar et al. show that increased intestinal permeability reduces hepatic de novo lipogenesis, affecting plasma membrane fluidity and lifespan of RBCs, and the resulting increase in iron levels promotes bacterial growth. This mechanism may explain the increased risk of sepsis associated with inflammatory bowel disease.

## Introduction

MUC2 mucin is the main component of the colonic mucus layer that separates gut microbiota from the single layer of mucosal epithelial cells^1^. In *Muc2*^−/−^ mice, colonic mucin depletion is associated with a marked decrease in intestinal barrier function and tight junction protein alterations and is a model to study ulcerative colitis (UC) in inflammatory bowel disease (IBD)^2^. An impaired mucus barrier in patients with IBD is similarly associated with high gut permeability^3^. Consequently, this defect can potentially result in increased intestinal penetrants reaching the liver via portal veins ensuing inflammation^4,5^.

Increased intestinal permeability develops into sepsis, which has been reported in both UC and Crohn’s disease (CD) patients^6,7^ but the detailed mechanism remains unknown. Given that colectomy is widely prevalent to treat IBD patients with abnormally high postoperative sepsis-related mortality rates^8–10^, sepsis in IBD is an important area which has been understudied. Gut inflammation in IBD can potentially leave liver functions compromised, which plays a central role in host defense against sepsis^11^. The liver acts as the master regulator of iron, which is required for oxygen transport, a cofactor for enzymatic functions and critical for controlling bacterial growth/inflammation^12^. Tight regulation of iron levels in the host is critical as low levels of iron cause anemia and excessive iron load is toxic due to the generation of free reactive oxygen species (via Fenton’s reaction) in addition to favoring pathogen survival^13^. The majority of iron acquisition in the host takes place through recycling and therefore dietary iron is minimally absorbed by duodenal enterocytes^14^. Red blood cells (RBCs) contains the majority of iron that is recycled by macrophage-mediated phagocytosis of senescent/aged RBCs^15^. Senescent RBCs membrane ruffling due to continuous passage through narrow capillaries exposes phosphatidyl serine, which is sensed by macrophages and phagocytose to recycle their contents^16^. For this reason, the life spans of RBCs are mainly determined by fluidity of their plasma membrane, which is maintained by proper ratio of saturated and unsaturated fatty acid levels^17^. Conversion from saturated fatty acid to mono-unsaturated fatty acid (MUFA) or poly-unsaturated fatty acids (PUFA) thus become extremely important for the maintenance of optimum membrane fluidity of RBCs^18^. MUFA is generated mainly by the action of stearoyl coenzyme A desaturase-1 (SCD-1) in hepatocytes and their expression during IBD has been shown to be reduced^19^. The consequence of this deficiency in IBD however, remains largely unknown. Erythrocyte membrane fluidity among IBD patients has been reported to be significantly lower than healthy individuals^20^. This could be attributed to altered lipid biosynthesis or fatty acid contents which in turn shorten the 120 days RBC life span^21,22^. Macrophages play an important role both in iron recycling via phagocytosing senescent RBCs and iron storage^14^.

As continuous exposure to gut microbial antigen triggers imbalance in pro-inflammatory and anti-inflammatory factors, we hypothesize it may affect erythrophagocytosis by macrophages. To investigate this, we have used *Muc2*^+/+^ and *Muc2*^−/−^ littermates to normalize their microbiota and to tease out the distinct effects of increased microbial penetrants in the absence of a mucus barrier. Herein, we report that baseline inflammation in IBD can affect membrane fluidity due to decreased hepatic lipogenesis, which in turn can potentially alter senescent erythrocytes clearance to increase circulatory iron levels to promote sepsis.

## Results

### *Muc2*^−/−^ littermates exhibit basal systemic inflammation

**Fig. 1:**
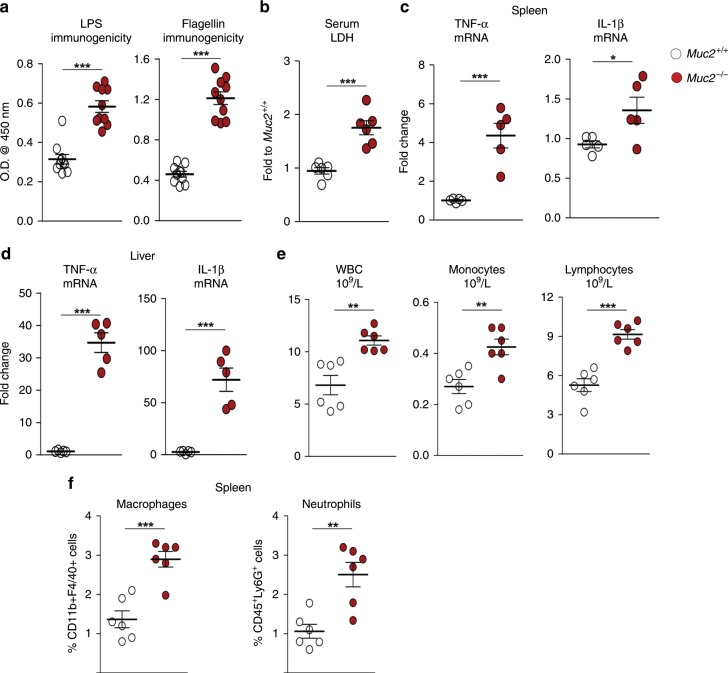
*Muc2*^−/−^ littermates exhibit basal systemic inflammation. **a** Determination of antibodies against LPS and flagellin in the sera of *Muc2*^−/−^ littermates by ELISA (*n* = 10). Representative data are from four independent experiments; paired Student’s *t* test. **b** High basal LDH levels in the circulation confirm damage and toxicity of tissue in *Muc2*^−/−^ littermates (*n* = 6). Representative data from three independent experiments; paired Student’s *t*-test. **c**, **d** High pro-inflammatory cytokine gene expression in the spleen and liver of *Muc2*^−/−^ littermates as determined by real time RT-PCR (*n* = 5). Representative data of four experiments; paired Student’s *t* test. **e** Complete blood count (CBC) analysis showing high levels of WBC, monocytes, and lymphocytes in the circulation of *Muc2*^−/−^ animals as markers of systemic inflammation (*n* = 6). Representative data from two different experiments; paired Student’s *t* test. **f** Percent macrophages and neutrophil population in spleen from naive *Muc2*^−/−^ and *Muc2*^+/+^ littermates. Single live cells population were gated for CD11b^+^F4/40^+^ (macrophages) and CD45^+^Ly6G^+^ neutrophils (*n* = 6). Representative data from two independent experiments. Abbreviations used: LPS lipopolysaccharide; LDH lactate dehydrogenase; TNF-α tumor necrosis factor alpha; IL-1β interleukin 1 beta; WBC white blood cells. Data are presented as means ± SEM. *p < 0.05, **p < 0.01, and ***p < 0.001.

Deficiency in Muc2 mucus caused constitutive low-grade inflammation characterized by significantly reduced body weight (BW) gain, increase colon weight, splenomegaly (Supplementary Fig. 1A–C) and increased susceptibility to DSS-induced colitis^23^. In addition, there were significantly higher levels of anti-LPS and anti-flagellin antibodies in *Muc2*^−/−^ littermates (Fig. 1a) suggesting increased bacterial sensing by the host. Additionally, there was a ~2-fold increase in serum lactate dehydrogenase (LDH) levels and higher TNF-α and IL-β pro-inflammatory mRNA expression in the spleen and liver (Fig. 1b–d) in *Muc2*^−/−^ but not in *Muc2*^*+/+*^ littermates. *Muc2*^−/−^ littermates also showed increase in WBC, monocytes, and lymphocytes in blood and significantly higher splenic macrophages and neutrophils suggesting ongoing systemic inflammation (Fig. 1e, f). *Muc2*^−/−^ dysfunctional mucosal epithelial barrier with high immune cell infiltration^24^ culminated in occasional rectal prolapse in 30–50% of littermates between 2 and 5 months of age^25^ (Supplementary Fig. 1D).

### *Muc2*^−/−^ mice are highly susceptibile to sepsis

**Fig. 2:**
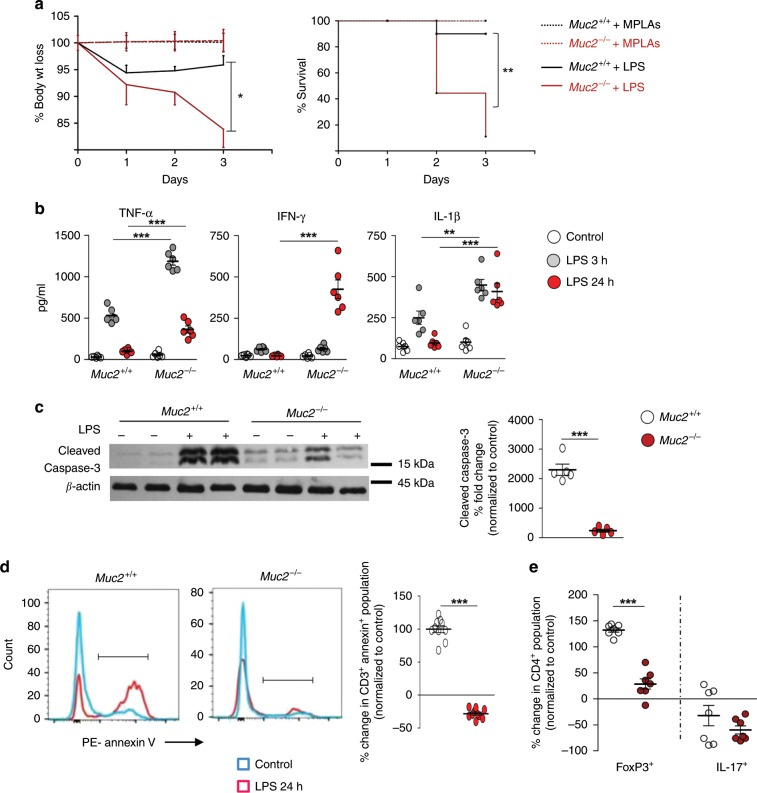
*Muc2*^−/−^ mice are highly susceptibility to sepsis. **a** Comparative sensitivities of *Muc2*^−/−^ littermates to sepsis following intraperitoneal administration of LPS at 5 mg/kg body weight. Untreated animals received a similar dose of monophosphoryl lipid A (MPLAs). LPS caused higher body weight loss and mortality in *Muc2*^−/−^ littermates (*n* = 8). Representative data from four independent experiments; paired one-way ANOVA. **b** Comparative analysis of pro-inflammatory cytokines levels at different time points in the blood following LPS challenge indicating unregulated heightened immune response in *Muc2*^−/−^ littermates (*n* = 6). Representative data are from three independent experiments: Student’s *t* test. **c** and **d** LPS inoculation in *Muc2*^−/−^ show reduced levels of apoptotic splenocytes. **c** Representative immunoblot from four independent experiments demonstrating that unlike *Muc2*^*+/+*^, splenocytes from *Muc2*^−/−^ littermates undergo attenuated apoptosis (cleaved caspase-3) 24 h post LPS challenge (*n* = 5). Quantitative analysis represents values normalized to untreated group (paired Student’s *t* test). **d** Representative flow cytometry histogram and cumulative analysis of splenic CD3^+^ T-cells apoptosis showing significantly lower populations of Annexin-V-positive cells in *Muc2*^−/−^ as compared to *Muc2*^+/+^ littermates 24 h post LPS (*n* = 10). Data are representative of three independent experiments, Student’s *t* test. **e** Reduced Tregs CD4 cell population in *Muc2*^−/−^ littermates following LPS challenge shows unregulated heightened immune response, whereas Th17 CD4 cell populations remains unchanged in both genotypes (*n* = 7). Data are representative of three independent experiments, Student’s *t* test. *p < 0.05, **p < 0.01, and ***p < 0.001.

To determine if ongoing systemic inflammation in *Muc2*^−/−^ increased their susceptibility towards sepsis, we used a toxemia model, where a single sub-lethal dose of LPS was injected intraperitoneally. This model was preferred over surgically induced sepsis as it results in rapid systemic response and presents fewer concerns to animal welfare^26^. Surprisingly, *Muc2*^−/−^ but not *Muc2*^*+/+*^ littermates lost BW rapidly following LPS challenge and by day 3, up to 90% mortality was observed (Fig. 2a). To determine if the absence of a Muc2 mucus layer was predisposing *Muc2*^−/−^ for increase risk for LPS-induced sepsis, persistent low-grade colitis that caused alterations/depletion of the mucus layer was induced in *Muc2*^+/+^ littermates by treating with 0.75% DSS for 3 weeks. Colitis was confirmed by attenuated BW gain during DSS treatment and increased gut permeability after 3 weeks (Supplementary Fig. 1E). At the end of treatment, animals injected intraperitoneally with LPS showed increased BW loss coupled with up to 40% mortality (Supplementary Fig. 1F). These data clearly implicate perturbation of the mucus layer in colitis is a major risk factor for sepsis similar to what is observed in *Muc2*^−/−^ littermates.

In *Muc2*^−/−^ littermates following LPS challenge, mortality was associated with high circulatory signature sepsis pro-inflammatory cytokines TNF-α, IFN-γ, and IL-1β as early as 3 h that failed to resolve after 24 h (Fig. 2b). We then determined if animals were immunosuppressed, where increased death of immune cells are known to be associated with elevated pro-inflammatory cytokine produced by the host^27^. We observed higher apoptosis in splenocytes from *Muc2*^+/+^ following LPS challenge as compared to untreated *Muc2*^−/−^ (Fig. 2c), which suggested immune-dysregulation in *Muc2*^−/−^ littermates. Further investigation revealed splenic CD3^+^ T-cells from *Muc2*^−/−^ but not from *Muc2*^+/+^ littermates were more resistant to apoptosis upon LPS challenge (Fig. 2d). This was associated with a concomitant reduction in CD4^+^FoxP3^+^ T-reg population in *Muc2*^−/−^ following LPS challenge as compared to *Muc2*^+/+^ littermates. However, there was no significant difference in CD4^+^IL-17^+^ cells between the two genotypes (Fig. 2e). Collectively, these results suggest unresponsiveness of *Muc2*^−/−^ immune cells, particularly T-cells, towards exogenous LPS challenge, potentially due to already high levels of LPS and other inflammatory microbial components in the circulation owing to a defective mucus barrier.

### Increased bacterial translocation in *Muc2*^−/−^ littermates

**Fig. 3:**
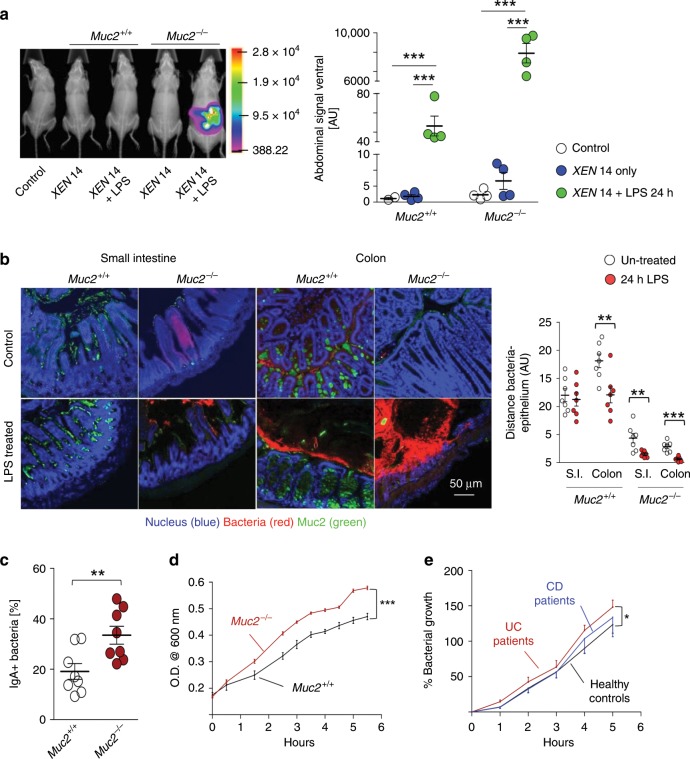
Increased bacterial translocation in *Muc2*^−/−^ littermates. **a** Bioluminescence of *XEN*-14 *E. coli* treated and control animals were recorded post 24 h LPS treatment. LPS-treated *Muc2*^−/−^ exhibited increased luminescence corresponding to higher translocation of intestinal bacteria as compared to *Muc2*^+/+^ littermates. Graph shows representative data from two independent experiments. **b** Fluorescence in situ hybridization (FISH) using Alexa 555-conjugated EUB338 universal probe for bacterial rRNA in mouse intestinal tissue 24 h post LPS challenge to visualize translocated gut bacteria. Tissues were fixed in Carnoy’s, paraffin embedded, sectioned, and FISH staining was done using established protocol. Nucleus was stained with DAPI and mucus with UEA1-TRITC lectin. Representative images are from three independent experiment, *n* = 3. Graph shows distance calculated from epithelium to the nearest bacteria (select straight line from epithelium to the bacteria and then analysis tab). In total 25 events were calculated from each area of view and plotted as Means ± SEM. **c** Quantification of fecal IgA^+^ bacteria in *Muc2*^−/−^ and *Muc2*^+/+^ littermates. Age and sex-matched animals were used to collect fecal samples in sterile condition. Graph shows cumulative data of two independent experiments (*n* = 5/experiment) and plotted as mean ± SEM. **d**, **e** Higher bacterial growth in the serum of **d** *Muc2*^−/−^ littermates (*n* = 10) and **e** active UC patients, suggesting higher predisposition towards septicemia (*n* = 5/group). A fixed number of non-pathogenic *E. coli* was added to heat-inactivated serum and O.D. was recorded at given time points at 600 nm. Data are representative of three independent experiments, paired one-way ANOVA. Data are presented as means ± SEM. *p < 0.05, **p < 0.01, and ***p < 0.001.

Exogenously administered LPS is known to affect intestinal permeability and to induce mucus secretion^28^. Consistent with this, we observed robust mucus secretion in the small intestine and colon in *Muc2*^+/+^ littermates following LPS treatment as revealed by Alcian blue staining and Muc2-specific antibody (Supplementary Fig. 2A, B). To determine if the lack of Muc2 mucin increased bacterial translocation and mortality in *Muc2*^−/−^ littermates, we used bioluminescent *E. coli XEN 14* and quantified bacterial translocation 24 h post LPS treatment. As predicted, in LPS-treated *Muc2*^−/−^ (Fig. 3a) the majority of the bioluminescence bacteria reached the liver and spleen as compared to *Muc2*^+/+^ littermates (Supplementary Fig. 2C). We also found high levels of endogenous Gram-negative bacteria (grown on MacConkey agar plates) residing in the mesenteric lymph nodes (MLNs) and spleen in *Muc2*^−/−^ following LPS treatment (Supplementary Fig. 2D). Intestinal bacteria in close proximity to the surface epithelium following LPS treatment were confirmed by florescence in situ hybridization (FISH) as compared to *Muc2*^+/+^ littermates (Fig. 3b). To investigate if intestinal bacteria breached the mucosal barrier to cause inflammation, we quantified the presence of IgA-coated bacteria^29^ in fecal pellets from *Muc2*^−/−^ and *Muc2*^+/+^ and observed significantly higher percentage of IgA+ bacteria in *Muc2*^−/−^ littermates (Fig. 3c). As bacterial presence in the circulation (septicaemia) is a hallmark of sepsis, we determined if bacteria could survive in the circulation of *Muc2*^−/−^ better than *Muc2*^+/+^ to cause increased damage. To do this, bacteria growth was determined in complement-inactivated serum that showed significantly increased bacterial growth in the serum of *Muc2*^−/−^ littermates (Fig. 3d). There was also higher bacterial growth in the serum of *Muc2*^+/+^ littermates after induction of chronic colitis by continuous low dose DSS (Supplementary Fig. 2E). Similar bacterial growth was observed in the serum from active UC patients as compared to healthy controls and CD patients (Fig. 3e). Overall, these data suggest that a dysfunctional mucus layer common in IBD patients can potentially exacerbates sepsis not only by allowing intestinal bacteria to translocate to systemic sites but also by modifying the systemic milieu to support higher bacterial growth.

### High circulatory iron in *Muc2*^−/−^ promotes bacteria survival

**Fig. 4:**
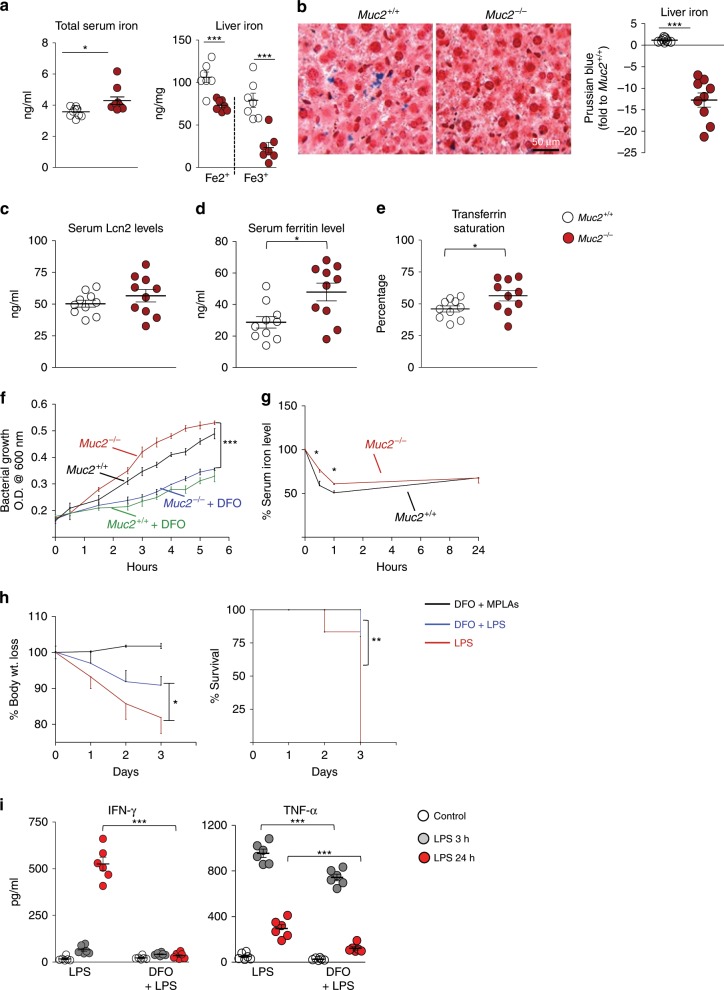
High circulatory iron in *Muc2*^−*/−*^ promotes bacteria survival. **a** Increased serum total iron levels and lowered Fe^2+^/Fe^3+^ iron levels in the liver of *Muc2*^−/−^ littermates suggests dysregulated iron homeostasis (*n* = 7–10). Sera were hemolysis free. Data are representative of four independent experiments, Student’s *t* test. **b** Liver samples were collected from saline perfused euthanized animals, formalin fixed, paraffin embedded, sectioned, and stained with Prussian blue for iron. Representative Prussian blue-stained liver samples confirm lower iron deposits in *Muc2*^−/−^ littermates (*n* = 4). Representative images are from four independent experiments. Means ± SEM, paired Student’s *t* test. **c** ELISA-based serum lipocalin 2 (NGAL) quantification from naïve *Muc2*^−/−^ and *Muc2*^+/+^ littermates (*n* = 10). Graph shows data from one of two independent experiments. **d** Serum ferritin levels quantification from *Muc2*^−/−^ and *Muc2*^+/+^ (*n* = 5) using ELISA. Graph shows cumulative data from two independent experiments. **e** Serum transferrin saturation levels from *Muc2*^−/−^ and *Muc2*^+/+^ animals (*n* = 5/experiment); cumulative data shown of two independent experiments. **f** Reduced bacterial growth in deferoxamine (DFO)-treated *Muc2*^−/−^ and *Muc2*^+/+^ littermates serum as compared to untreated control (*n* = 4). Representative of three independent experiments; paired one-way ANOVA. **g** Blood samples were collected following LPS challenge at the indicated time points and subjected to total iron quantification using hemolysis-free serum (*n* = 5). Delayed hypoferremia was recorded in *Muc2*^−/−^ littermates. Representative data are from three experiments, paired one-way ANOVA. **h** Comparative susceptibility to sepsis in *Muc2*^−/−^ littermates treated or not with DFO and challenged with 5 mg/kg body weight LPS intraperitoneally. Animals treated with DFO showed reduced weight loss and increased survival compared to the untreated group (*n* = 6–8). Representative data are from four independent experiments; paired one-way ANOVA. **i** Comparison of pro-inflammatory cytokines levels in the blood following LPS challenge in DFO treated and untreated *Muc2*^−/−^ littermates indicate unregulated attenuated immune response (*n* = 6). Representative data are from three independent experiments; paired Student’s *t* test. *p < 0.05, **p < 0.01, and ***p < 0.001.

We next explored the host factors that can support/potentiate bacterial growth in the circulation. Excess iron in known to promote bacterial growth in blood and may increase susceptibility towards sepsis^30^. Consistent with this, we observed at basal state, *Muc2*^−/−^ had significantly higher (up to 1.5-fold) total iron in the serum and lower levels of Fe^2+^/Fe^3+^ in the liver as compared to *Muc2*^+/+^ littermates (Fig. 4a). The presence of lower iron levels in *Muc2*^−/−^ mice liver was confirmed by Prussian’s iron staining (Fig. 4b). We found a similar trend of higher circulatory iron levels in *Muc2*^+/+^ littermates after induction of long-term colitis with low dose DSS (0.75%) for 3 weeks; however total iron levels in the liver was not significant (Supplementary Fig. 3A). The higher iron levels in the circulation was not due to increased absorption of dietary iron as the uptake of orally administered ferrous sulfate by *Muc2*^−/−^ and *Muc2*^*+/+*^ littermates showed similar circulatory iron levels measured up to 24 h (Supplementary Fig. 3B). Similarly, we also observed comparable transcript levels of the iron regulator genes, hepcidin, ferroportin, and DMT-1 in the liver between both genotypes (Supplementary Fig. 3C). There was also comparable levels of Zip-14 transcripts in the duodenum, splenocytes, and liver of *Muc2*^−/−^ and *Muc2*^*+/+*^ (Supplementary Fig. 3D) suggesting similar efficiency in iron absorption by both genotypes^31^. We next investigated the levels of serum lipocalin2 as they are known to alleviate iron over-load and disposal of iron from the host^32^ and found comparable levels in the circulation of both genotypes (Fig. 4c). To further investigate iron dysregulation in *Muc2*^−/−^, we quantified serum ferritin and transferrin saturation levels and found higher serum ferritin and transferrin saturation levels in *Muc2*^−/−^ but not in *Muc2*^+/+^ littermates (Fig. 4d, e). Ferritin is regarded as an acute phase protein that is released in the circulation from damaged hepatocytes, thus high serum ferritin levels could suggest abnormal liver function in *Muc2*^−/−^ animals^33^. As expected, excess iron in the serum of *Muc2*^−/−^ promoted bacterial growth that was attenuated following chelation with deferoxamine (DFO; Fig. 4f). To confirm iron chelation-dependent mechanism of DFO, we quantified serum iron levels of DFO-treated animal (3 h post DFO treatment) and observed significantly reduced iron levels (Supplementary Fig. 3E).

To establish a correlation between excess iron and mortality, we measured iron levels following LPS treatment and found that *Muc2*^−/−^ experience delayed hypoferremia as compared to *Muc2*^+/+^ littermates (Fig. 4g, Supplementary Fig. 3F). Moreover, we did not find significant differences between the key iron-regulating genes from the liver (hepcidin), duodenum, and spleen (Zip-14) (Supplementary Fig. 3G). To confirm in vivo if excess circulatory iron in *Muc2*^−/−^ could be the cause of accelerated mortality, animals were pre-treated with DFO 2 h before LPS challenge that significantly rescued animals against BW loss and mortality (Fig. 4h) and resulted in attenuated levels of serum pro-inflammatory cytokines (Fig. 4i, Supplementary Fig. 3H). Overall these results suggest ongoing inflammation due to increased intestinal permeability dysregulate iron levels that could exacerbate sepsis.

### Increased RBC membrane fragility in *Muc2*^−/−^ littermates

**Fig. 5:**
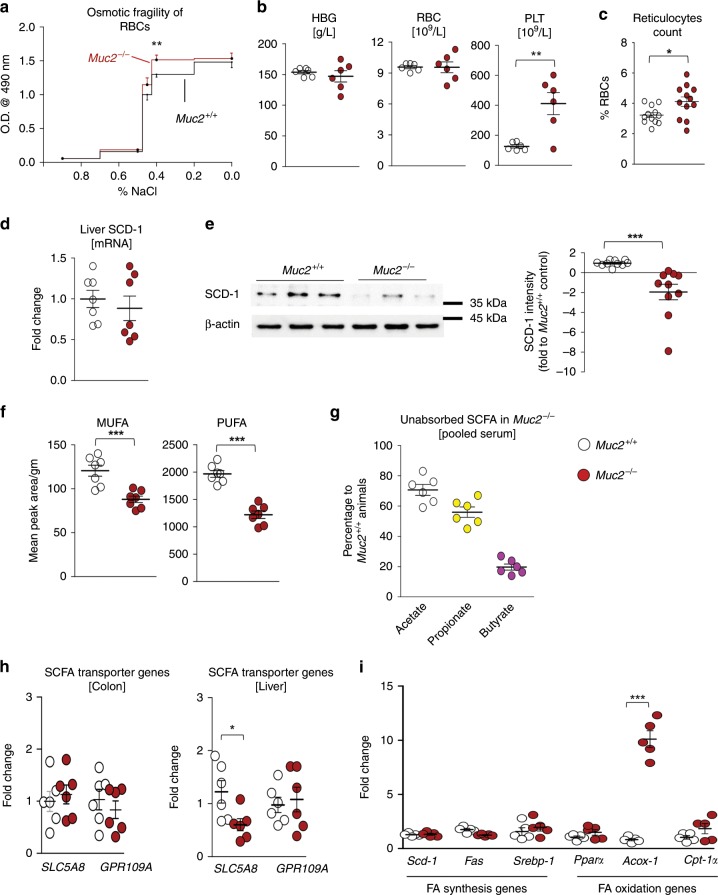
Increased RBC membrane fragility of *Muc2*^−*/*−^ littermates. **a** Increased basal osmotic fragility of RBCs isolated from *Muc2*^−/−^ littermates when subjected to varying concentration of NaCl. Representative data are from three independent experiments (*n* = 4–7), paired one-way ANOVA. **b** Complete blood count (CBC) analysis indicates no significant reduction in hemoglobin (HGB) levels and RBCs count in *Muc2*^−/−^ as compared to *Muc2*^*+/+*^ littermates (*n* = 6). Higher platelets (PLT) count in *Muc2*^−/−^ however indicates increased hemolysis. Representative data are from three independent experiments, paired Student’s *t* test. **c** Percent reticulocytes count in the blood from *Muc2*^−/−^ and *Muc2*^+/+^ littermates using new methylene blue dye (*n* = 5). Representative data from one of two independent experiments. **d**, **e** Levels of hepatic SCD-1 **d** mRNA (*n* = 7) and **e** protein levels in *Muc2*^−/−^ at basal state suggest reduced activity as compared to *Muc2*^*+/+*^ littermates (*n* = 3–5). Graph shows cumulative data are from three independent experiments, paired Student’s *t* test. **f** Lowered levels of hepatic mono-unsaturated fatty acids (MUFA) and polyunsaturated fatty acids (PUFA) in *Muc2*^−/−^ as compared to *Muc2*^*+/+*^ littermates (*n* = 3–5). MUFA and PUFA levels were measured by ^1^H NMR in the lipophilic fraction collected after methanol and chloroform extraction of homogenized liver. Representative data are from three independent experiments, paired Student’s *t* test. **g** Percentage of SCFA present in the circulation of *Muc2*^−/−^ compared to *Muc2*^+/+^ littermates as measured by GC–MS analysis (*n* = 6–8). Representative data are from two experiments. **h** Relative quantification of gene transcripts levels involved in SCFA absorption (*SLC5A8* and *GPR109A*) in colonic tissue and liver samples from *Muc2*^−/−^ and *Muc2*^*+/+*^ littermates (*n* = 6). **i** Relative quantification of transcripts levels of key genes involved in fatty acid synthesis and fatty acid oxidation in liver samples from *Muc2*^−/−^ and *Muc2*^*+/+*^ mice (*n* = 5). Graph shows representative data from two independent experiments. *p < 0.05, **p < 0.01 and ***p < 0.001.

As most of the iron in RBCs is recycled by splenic macrophages, we investigated RBC recycling capacity in *Muc2*^−/−^. We first determined the health of RBCs by measuring osmotic fragility of plasma membrane and found *Muc2*^−/−^ RBCs were significantly more fragile than those from *Muc2*^+/+^ littermates (Fig. 5a, Supplementary Fig. 4A, B). However, hemoglobin levels and RBC counts were comparable in both genotypes (Fig. 5b). An interesting finding was that *Muc2*^−/−^ had 2–3 fold higher platelet levels (thrombocytosis) (Fig. 5b), which is a strong indicator of hemolysis or ongoing infection and/or iron deficiency^34^. Accordingly, reticulocytes levels were quantified to investigate if erythropoiesis rate was higher as *Muc2*^−/−^ animals exhibited similar levels of RBC count despite increased osmotic fragility of RBCs. Predictably we observed significantly more reticulocytes in the circulation of *Muc2*^−/−^ as compared to *Muc2*^+/+^ littermates (Fig. 5c).

Increased fragility of RBCs could be due to decreased fluidity of plasma membrane,^35^ which is determined by the presence of unsaturated fatty acids^36^. Accordingly, we measured SCD-1 enzyme levels in the liver, a key molecule in the generation of MUFA. There were no significant differences at the transcript level (Fig. 5d), however protein expression was significantly downregulated in *Muc2*^−/−^ as compared to *Muc2*^+/+^ littermates (Fig. 5e). This suggests post transcription regulation of the SCD-1 gene in *Muc2*^−/−^. We also measured hepatic SCD-1 transcript levels 24 h post LPS challenge and observed significant reduction in *Muc2*^−/−^ as compared to *Muc2*^+/+^ littermates (Supplementary Fig. 4C). Quantification of the relative abundance of hepatic unsaturated fatty acids by nuclear magnetic resonance (NMR), showed significantly decreased levels of MUFA and PUFA in *Muc2*^−/−^ as compared to *Muc2*^+/+^ (Fig. 5f, Supplementary Fig. 4D). Abdominal fat pad and serum triglycerides levels were also significantly decreased in *Muc2*^−/−^ (Supplementary Fig. 4E, F) that could be another indicator of reduced hepatic de novo lipogenesis. The main substrate for hepatic fatty acid synthesis is short-chain fatty acid (SCFA) produced by microbial fermentation in the cecum, which was found to be less in *Muc2*^−/−^ as measured by GC–MS (Supplementary Fig. 4G) and corroborates other studies^25^. Surprisingly, we observed higher levels of SCFAs in the serum of *Muc2*^−/−^ suggesting reduced absorption by the liver (Fig. 5g). To investigate this further, we quantified transcript levels of key SCFA transporter genes (*SLC5A8* and *GPR109A*) in the liver and colon tissues of naïve *Muc2*^−/−^ and *Muc2*^+/+^ littermates^37^. We observed comparable levels of SCFA transporter transcripts in colon, however hepatic *SCLC5A8* transcript levels were downregulated in *Muc2*^−/−^ animals (Fig. 5h). Finally, to understand the mechanism behind reduced lipid levels in *Muc2*^−/−^, we quantified transcript levels of key enzymes involved in hepatic FA synthesis and FA oxidation by real time RT-PCR. We observed comparable levels of genes involved in FA synthesis (*Scd-1*, *Fas*, and *Srebp-1*), however *Acox-1* transcript levels (involved in FA oxidation) was significantly upregulated in *Muc2*^−/−^ as compared to *Muc2*^+/+^ littermates (Fig. 5i). Taken together, these results suggest increased intestinal permeability cumulates into higher systemic inflammation that could attenuate hepatic lipid synthesis in *Muc2*^−/−^ littermates.

### Reduced erythrophagocytosis by *Muc2*^−/−^ macrophages

**Fig. 6:**
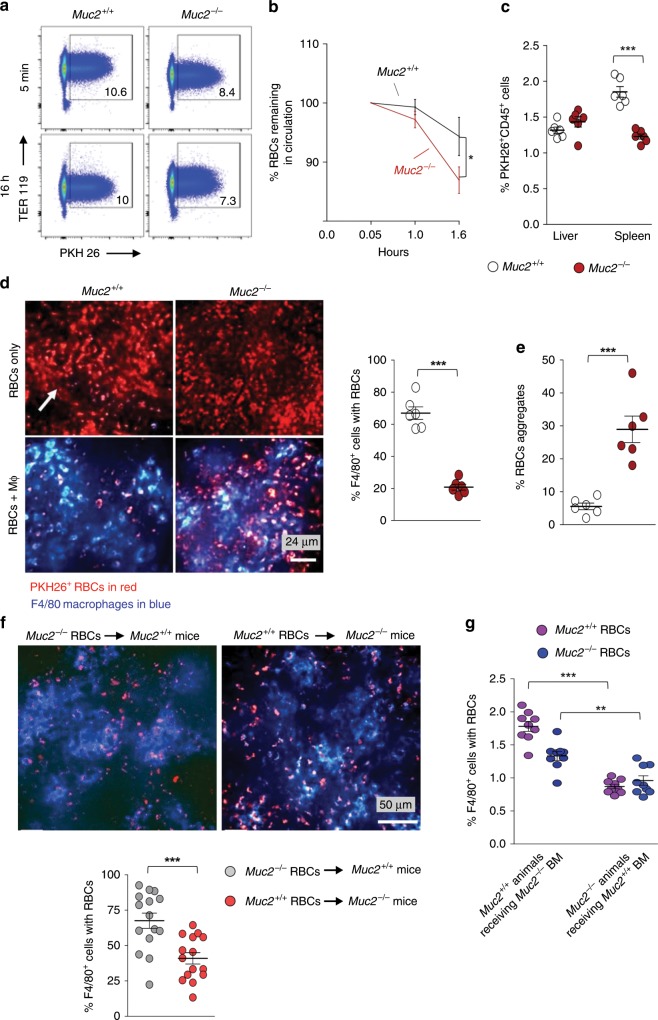
Reduced erythrophagocytosis by *Muc2*^−/−^ macrophages. **a**, **b** Erythrocytes from *Muc2*^−/−^ and *Muc2*^*+/+*^ littermates were isolated, labeled with a lipophilic dye PHK26 ex vivo and injected back into congenic recipients. Blood was drawn from PHK26^+^ erythrocytes recipient animals at the indicated time points and RBCs were stained with the erythrocyte marker Ter119 to identify PKH26^+^ RBCs. **a** Representative dot plots (one of three experiments) showing remaining labeled RBCs in the circulation after 16 h (*n* = 6) and **b** quantification showing percentage of PKH26^+^TER119^+^CD45^−^ cells remaining in the circulation at indicated time points (*n* = 6). Data are representative from three independent experiments. **c** Distribution of PKH26^+^CD45^+^ leukocytes in the spleen after 16 h post labeled RBCs challenge showing uptake of erythrocytes by these cells. Cells were gated as CD45^+^CD3^−^CD19^−^NK1.1-leukocytes in the spleen. Data represents three independent experiments, means ± SEM (*n* = 5 per group). **d** Representative data (one of three experiments) on distribution of PKH26^+^F4/80^+^ macrophages in spleen 16 h post labeled RBCs injection in mice as visualized by a Quorum WaveFx spinning disk confocal upright microscope. Labeled RBCs from one genotype were injected in the same genotype of mice (homologous group) can be seen in circulation (arrow, upper left panel) in *Muc2*^+/+^ spleen as compared to *Muc2*^−/−^ animals where they seem to be out of the circulation. Quantitative analysis of percentage of PKH26^+^F4/80^+^ cells in spleen suggests inefficient erythrophagocytosis in *Muc2*^−/−^ spleen. Means ± SEM (*n* = 5), paired Student’s *t* test. **e** Quantitative analysis of RBCs aggregates in the spleen suggests significantly higher proportion of senescent RBCs were not phagocytosed in *Muc2*^−/−^ littermates (*n* = 5–8). **f** Representative images showing presence of macrophages in spleen after heterologous transfer (RBCs from *Muc2*^+/+^ were transferred into *Muc2*^−/−^ littermates and vice versa) with labeled RBCs. Quantitative analysis of percentage of PKH26^+^F4/80^+^ cells show inefficient clearance of *Muc2*^+/+^ RBCs in *Muc2*^−/−^ spleen (*n* = 5–8). **g** Quantitative analysis of PKH26^+^ RBCs uptake by splenic macrophages in bone-marrow chimeras. Data represents two independent experiments. Means ± SEM (*n* = 3), paired Student’s *t* test. *p < 0.05, **p < 0.01, and ***p < 0.001.

Splenic macrophages recognize and phagocytose senescent/damaged RBCs in the circulation to recycle its iron contents^14^. However, the presence of pro-inflammatory cytokines may induce functional changes in macrophages that could impede erythrophagocytosis. To investigate if erythrophagocytosis was impaired in *Muc2* littermates, RBCs from each genotype was isolated and labeled with a stable lipophilic dye PKH26 that incorporates into >90% RBCs and injected back into each respective littermates (see flow chart and gating strategy in Supplementary Fig. 5A, B). Remarkably, clearance of RBCs from the circulation in *Muc2*^−/−^ was significantly higher as compared to *Muc2*^+/+^ littermates (Fig. 6a, b). This suggests *Muc2*^−/−^ RBCs may have reduced life span and/or cleared faster from the circulation by the spleen. Accordingly, we quantified splenic CD45^+^PKH26^+^ cells by FACS and found their populations were significantly less in *Muc2*^−/−^ than *Muc2*^+/+^ littermates (Fig. 6c). This suggests that splenic leukocytes in *Muc2*^−/−^ were not clearing RBCs that leave the circulation. In support of this, we found little iron deposits in the spleen of *Muc2*^−/−^ by Prussian blue staining (Supplementary Fig. 5C). Taken together with the in vitro data where RBCs were shown to be more fragile with high platelet counts (Fig. 5a, b), these data suggests increased hemolysis might explain the high iron load in the circulation of *Muc2*^−/−^ littermates. To further investigate erythrophagocytosis events in the spleen, we visualized PKH26^+^RBCs using Quorum WaveFx spinning disk confocal upright microscope after 16 h post-transfer. *Muc2*^*+/+*^ RBCs remained in the circulation (Fig. 6d, brighter arteries indicated with arrow) whereas *Muc2*^−/−^ RBCs were scattered throughout the spleen supporting the FACS data where *Muc2*^−/−^ RBCs left the circulation faster. Next, we injected fluorochrome-labeled anti-mouse F4/80 antibodies to visualize erythrophagocytosis efficiency and found less erythrophagocytosis events in *Muc2*^−/−^ (Fig. 6d). Strikingly, senescent RBCs that were not cleared/recycled by splenic macrophages were found clustered together and formed aggregates in the spleen of *Muc2*^−/−^ littermates (Fig. 6e). To establish if basal inflammation in *Muc2*^−/−^ impaired erythrocyte recycling efficiency, PKH26-labeled *Muc2*^+/+^ RBCs were injected into *Muc2*^−/−^ littermates and vice versa. Interestingly, we observed that *Muc2*^*+/+*^ splenic macrophages phagocytosed *Muc2*^−/−^ RBCs as efficiently as homologous controls (*Muc2*^+/+^ receiving *Muc2*^+/+^ RBCs; Fig. 6f). In contrast, *Muc2*^−/−^ splenic macrophages were less efficient in clearing *Muc2*^*+/+*^ RBCs (Fig. 6f). These results indicate that erythrophagocytosis by *Muc2*^−/−^ macrophages were compromised leading to excessive iron levels in the circulation. To rule out side effects of genetic deficiency in *Muc2*^−/−^ on erythrophagocytosis, we generated bone marrow chimeras (Supplementary Fig. 5D) using *Muc2*^−/−^/*Muc2*^+/+^ littermates. Thereafter, we transferred PKH26-labeled RBCs isolated from untreated naïve *Muc2*^−/−^/*Muc2*^+/+^ animals. Expectedly, we found that erythrophagocytosis efficiency was restored in macrophages isolated from *Muc2*^*+/+*^ receiving *Muc2*^−/−^ bone marrow as compared to the control group *Muc2*^−/−^ receiving *Muc2*^−/−^ bone marrow (Fig. 6g). We also observed reduced levels of erythrophagocytosis in *Muc2*^−/−^ receiving *Muc2*^*+/+*^ BM (Fig. 6g). *Muc2*^−/−^ receiving *Muc2*^*+/+*^ bone marrow exhibited high serum total iron and LDH levels (Supplementary Fig. 6A, B). However, osmotic fragility index was comparable between both groups (Supplementary Fig. 6C). We also observed significantly increased bacterial growth in *Muc2*^−/−^ receiving *Muc2*^*+/+*^ bone marrow as compared to *Muc2*^*+/+*^ receiving *Muc2*^−/−^ BM (Supplementary Fig. 6D). Finally, *Muc2*^−/−^ receiving *Muc2*^*+/+*^ bone marrow exhibited higher susceptibility towards LPS-induced sepsis and mortality than *Muc2*^*+/+*^ littermates receiving *Muc2*^−/−^ bone marrow (Supplementary Fig. 6E). Overall, these data indicate that decreased erythrophagocytosis in *Muc2*^−/−^ littermates could be associated with increased intestinal permeability that potentially led to the generation of an iron-rich milieu.

### Antibiotics treatment attenuates sepsis in *Muc2*^−/−^

**Fig. 7:**
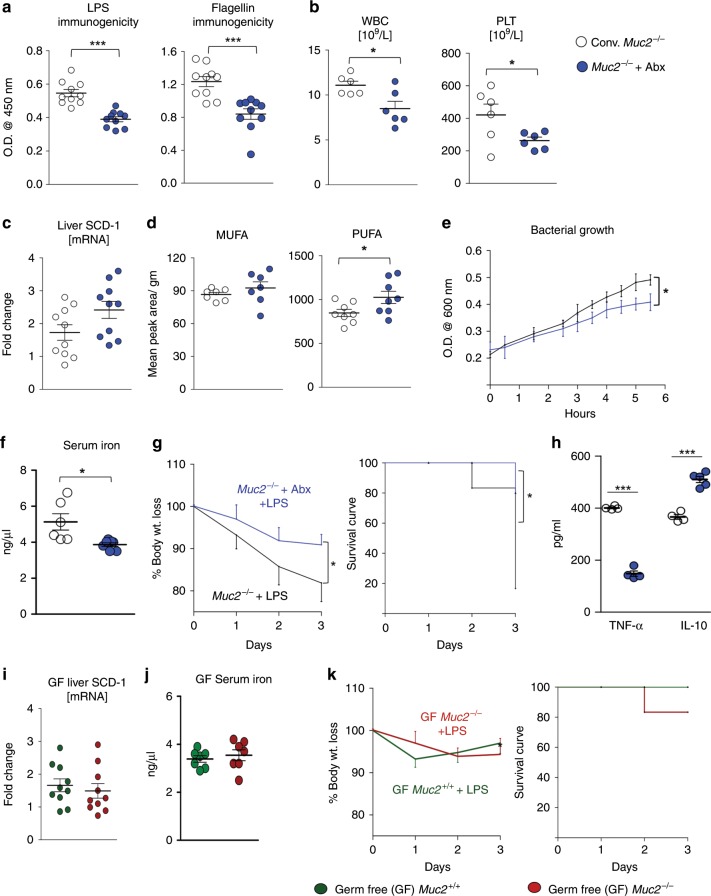
Antibiotics treatment attenuates sepsis in *Muc2*^−/−^. **a** Antibiotics (Abx)-treated *Muc2*^−/−^ animals shows a reduction in antibodies against LPS and flagellin as compared to the untreated group (*n* = 10). Representative data are from four independent experiments; paired Student’s *t* test. **b** Complete blood count (CBC) analysis on Abx-treated *Muc2*^−/−^ animals indicates significant reduction in WBC (reduced inflammation) and platelets count (lowered hemolysis) in comparison to the untreated group. *n* = 6 per group, representative data are from two independent experiments, paired Student’s *t* test. **c**, **d** Hepatic SCD-1 mRNA levels **c** in Abx-treated *Muc2*^−/−^ animals shows increase but non-significant expression/restoration of fatty acid biosynthesis; *p*-value = 0.065 (*n* = 6). Production of MUFA and PUFA levels as analyzed by NMR (**d**) were not increased after Abx treatment suggesting critical role of SCFA produced by gut microbial fermentation. Representative data are from two independent experiments, *n* = 5 each group, paired Student’s *t* test. **e** Reduction in bacterial growth in serum isolated from Abx-treated *Muc2*^−/−^ as compared to untreated *Muc2*^−/−^ littermates. **f** Attenuated levels of total iron content in circulation was observed in Abx-treated *Muc2*^−/−^ animals. *n* = 6 per group, representative data from two independent experiments, paired Student’s *t* test. **g** Reduced sepsis index in Abx-treated *Muc2*^−/−^ after intraperitoneally LPS challenge suggests reduction in basal inflammation can decrease susceptibility to sepsis induce morbidity and mortality in *Muc2*^−/−^ littermates. *n* = 8 per group, representative data from four independent experiments; paired one-way ANOVA. **h** Attenuated production of pro and anti-inflammatory cytokines in Abx-treated *Muc2*^−/−^ animals as compared to the untreated group. *n* = 5 per group, representative data are from four independent experiments; paired Student’s *t* test. **i**–**k** Germ-free *Muc2*^−/−^ and *Muc2*^+/+^ mice were treated with LPS to induce septic shock. We observed comparable transcript levels of SCD-1 gene **i** and total iron content in circulation **j** in both germ-free genotypes (*n* = 6–10). **k** Representative data showing body weight loss and survival index following LPS challenge (5 mg/kg body weight) in germ-free *Muc2*^+/+^ and *Muc2*^−/−^ mice (*n* = 5). *p < 0.05, ***p < 0.001.

To determine if increased basal inflammation in *Muc2*^−/−^ were the cause of dysregulated iron homeostasis and attenuated lipid levels, animals were treated with a broad-spectrum antibiotic (Abx) cocktail. Abx treatment decreased the levels of antibodies against LPS and flagellin (Fig. 7a) and lowered TNF-α mRNA expression in the spleen and liver of *Muc2*^−/−^ as compared to untreated *Muc2*^−/−^ littermates (Supplementary Fig. 7A). Reducing inflammation lowered WBC, platelets (Fig. 7b), monocytes and lymphocytes (Supplementary Fig. 7B) in the circulation. This resulted in increased serum triglyceride (Supplementary Fig. 7C) hepatic SCD-1 transcript levels (although not significant, *p*-value = 0.059; Fig. 7c) and hepatic PUFA levels (Fig. 7d). Following Abx treatment, RBCs fragility score improved (Supplementary Fig. 7D) that attenuated growth of bacteria in the serum (Fig. 7e) partially due to significantly lowered circulatory total iron levels (Fig. 7f). Consequently, susceptibility towards sepsis reduced dramatically as evidenced by improved survival index (Fig. 7g) and attenuated levels of TNF-α, a signature pro-inflammatory cytokine, following LPS challenge (Fig. 7h). To substantiate these findings, we used germ-free (GF) *Muc2*^+/+^ and *Muc2*^−/−^ animals and observed comparable levels of hepatic SCD-1 transcripts (Fig. 7i) and total serum iron levels (Fig. 7j), however there was no significant difference in susceptibility towards LPS-induced septic shock (Fig. 7k).

## Discussion

The intestinal mucus layer prevents gut microbiota from gaining access to host tissues to trigger inflammation^1^. IBD patients with a defective intestinal barrier are at higher risks for bacterial translocation and exposure to gut microbial antigens, which when exceed the tolerance/clearance threshold of immune cells, may lead to sepsis^6,7^. Despite circumstantial evidence that sepsis is due to gut-derived antigens/organisms, there is a paucity of clinical studies directly implicating intestinal bacterial translocation as an important risk factor^38–41^. Sepsis is commonly defined as unregulated immune response towards microbial antigens that is a leading cause of death worldwide^42,43^. Sepsis-related death is particularly high in post-operative IBD patients^8–10^. With increasing incidence of IBD worldwide and lack of relevant studies done on sepsis in IBD patients^6,7^, this study was conducted using *Muc2*^−/−^ animals as a murine model for IBD. *Muc2*^−/−^ animals lack an adherent intestinal mucus layer and exhibit a leaky gut phenotype that is a hallmark for IBD^23^. Muc2 mucin is produced and secreted by intestinal goblet cells and *Muc*2 gene deficiency does not affect other cell type function. For these reasons *Muc2*^−/−^-deficient mice were used in this study versus other animal models of colitis that are either chemical induced or immunodeficiency based.

Here we show that *Muc2*^−/−^ littermates are high risks for developing sepsis following a sub-lethal dose of exogenous LPS. Immune dysregulation in sepsis determines the outcome of the disease. In spite of recent advances in the field of sepsis immunobiology and therapeutics development, mortality rate is alarmingly high^27^ suggesting multiple ways of immune system activation during sepsis. Increased immune cells apoptosis is commonly seen in sepsis as a sign of immunosuppression, which can lead to establishment of infection and eventual death^27,44^. An alternative theory suggest that organ failure/mortality during sepsis could be due to cellular stress leading towards “hibernation” of the cell to prevent against excessive apoptosis^45,46^. During this period, cells survive without performing tissue-specific roles. In support of this there are reports of reduced apoptosis of splenocytes from post mortem of sepsis patients^27^. In our study we observed decreased immune cells apoptosis in *Muc2*^−/−^, which may have resulted from continuous exposure to microbial antigen, leading towards overwhelming production of unresolved pro-inflammatory cytokine (septic shock) and mortality. We also observed decreased T-regs following LPS challenge in *Muc2*^−/−^ as compared to *Muc2*^*+/+*^ littermates. We believe this could be due to higher basal levels of circulating IL-6 in *Muc2*^−/−^ that could suppress the Treg phenotype^47^. Tregs are required for recovery from sepsis^48^ and can down regulate pro-inflammatory cytokine levels. There was increased penetration of intestinal bacteria into systemic site in *Muc2*^−/−^ animals following LPS challenge. Basally *Muc2*^−/−^ animals showed high percentage of fecal IgA^+^ bacteria suggesting the presence of inflammatory commensals that triggered intestinal inflammation. These finding also supports earlier reports where the presence of IgA^+^ bacteria uniquely identified colitogenic microbiota in a mouse model as well as in IBD patients^29^.

RBCs are the most abundant cell in the host and they die at a rapid rate of 2–3 million per second. Their life span is heavily affected with decrease in lipids as it can reduce fluidity^22^. RBCs are laden with iron as a component of hemoglobin and loss of hemoglobin/RBCs can cause anemia. Therefore, RBCs membrane integrity is of paramount importance. Previous studies have confirmed that RBCs membrane integrity are compromised in IBD^20–22^. RBCs membrane flexibility allows them to pass through narrow capillaries and the presence of lipid contents in plasma membrane ensures fluidity and permeability. Hepatic SCD-1 is the key enzyme active in hepatocyte that synthesizes MUFA. Its activity however can be altered with diet, microbiota, inflammation, and other gastrointestinal disease conditions and may affect the synthesis of unsaturated fatty acids. To date, no studies have described the effect of increased intestinal permeability in IBD on hepatic lipid biosynthesis in conjugation with iron homeostasis. The liver detoxifies blood and absorbs nutrients that it receives via hepatic portal vein, mixed with nutrients and microbial components. In GI barrier dysfunction the liver could be overloaded with increased microbial penetrants. Based on this hypothesis, we set our approach to find a link between GI disorders and metabolic functions of the liver that may alter iron homeostasis. We found that increased microbial penetrants in *Muc2*^−/−^ mice resulted in elevated systemic pro-inflammatory cytokines levels that in turn impart a dramatic effect on hepatic fatty acid biosynthesis. Liver SCD-1 enzyme plays a critical role in the generation of MUFA and PUFA, which are major components of plasma membrane phospholipids, providing them necessary fluidity^49^. Several studies have established a role for SCD-1 in promoting hepatic steatosis, insulin resistance, and obesity^50,51^. *Muc2*^−/−^ animals expressing lesser amount of SCD-1 may protect them from NAFLD^52^. However, less MUFA or PUFA levels could also decrease fluidity of plasma membrane and make them more fragile. RBCs are most affected as they pass through narrow capillaries and a rigid plasma membrane may decrease RBCs life span considerably, which is normally 120 days. Interestingly, lipids can modulate phagocytosis functions of macrophages as evidenced by a recent study using lipid emulsion of omega-3 fatty acid (PUFA) against experimental sepsis^53^. Earlier reports have been controversial regarding the beneficial effect of lipids, mainly because of insufficient data and lack of mechanistic evidence^54,55^.

The host acquire most of its iron needs through RBCs recycling mainly by splenic macrophages via erythrophagocytosis^15^, otherwise intravascular hemolysis would increase circulatory iron levels and aid in sepsis. Phagocytotic activity of macrophages under chronic conditions is severely affected in a pro-inflammatory cytokine-rich environment. One study have described a link between host iron regulation, disease activity, and pro-inflammatory cytokines in children with active CD^56^. In *Muc2*^−/−^ animals, splenic macrophages were less efficient in sensing and clearing senescent RBCs that resulted in higher iron load in the circulation. However, we cannot rule out the possibility that *Muc2*^−/−^ macrophages may also have altered efficiency in sensing senescent RBCs other than reduced erythrophagocytosis ability. In summary, our results have shown that prolonged low-grade inflammation caused by intestinal penetrants down regulated hepatic SCD-1 enzyme activity reducing RBCs membrane fluidity. Inflammation on the other hand hampered erythrophagocytosis by splenic macrophage increasing serum iron levels and susceptibility towards sepsis.

## Methods

### Ethics statement

The Health Sciences Animal Care Committee from the University of Calgary, have examined the animal care and treatment protocol (AC14-0219) and approved the experimental procedures proposed and certifies with the applicant that the care and treatment of animals used was in accordance with the principles outlined in the most recent policies on the “Guide to the Care and Use of Experimental Animals” by The Canadian Council on Animal Care. GF mice were obtained from the International Microbiome Centre at the University of Calgary. Human sample collection and usage were approved by the Conjoint Health Research Ethics Board of the University of Calgary (Control ID: REB14-2429). All research subjects consented to participate in this study before tissue collection and chart review.

### Animal treatment and reagents

Sepsis was induced by intraperitoneal injection of LPS (5 mg/kg BW; Supplementary Table 1) and observed for 3 days for BW loss, disease symptoms, and mortality. Un-treated animals received a similar dose of monophosphoryl lipid A (MPLA), a low toxicity derivative (dephosphorylated) of LPS. For dietary iron absorption assay, ammonium ferrous sulfate was dissolved in tap water, gavaged once daily as dose of 2 mg/kg and iron was measured in serum at different time points. For *E. coli*-*XEN*14 translocation experiment, animals were gavaged with 300 µl of overnight (~14 h) grown culture (OD600 adjusted to 1.25) daily for 3 days and challenged with LPS at the end of treatment. Signals were recorded after 24 h LPS challenge by using an optical imaging system in vivo extreme 4MP (Bruker, USA). Mice were treated with antibiotics according to the protocol described previously^57^. Briefly, animals were gavaged daily for 21 days with the following antibiotics cocktail: amphotericin-B (1 mg/kg), vancomycin (50 mg/kg), neomycin (100 mg/kg), and metronidazole (100 mg/kg). Additionally, ampicillin (1 mg/ml) was provided with drinking water. For chronic colitis, 0.75% dextran sulfate sodium salt (DSS) was administered to *Muc2*^*+/+*^ animals for 3 weeks. Intestinal permeability was measured by FITC dextran^23^.

### Serum LDH, ELISA, and iron assay

All assays were performed using hemolysis-free sera, which were collected by centrifugation of blood in serum separator tubes (BD Biosciences, USA). Serum LDH levels were determined by colorimetric LDH assay kit (Abcam, ON, Canada) as per manufacturer’s protocol. Serum lipocalin 2 was detected and quantified using mouse lipocalin 2 duoset ELISA kit (R&D systems; Catalog No. DY1857-05). Levels of anti-flagellin and LPS antibodies in serum was detected by ELISA^58^. Serum cytokine levels were measured using luminex (Eve technologies, University of Calgary, Calgary). Total iron content was quantified using hemolysis-free sera and from saline-perfused liver samples from animals. Iron quantification kit (Sigma, USA) was used to determine total iron content following manufacturer’s protocol.

### Ferritin quantification and transferrin saturation assay

Serum ferritin level quantification was performed using mouse ferritin ELISA kit (Abcam) following manufacturer’s protocol. Total iron-binding capacity (TIBC) or transferrin saturation was quantified using TIBC kit (Randox; TI1010) and serum iron quantification kit (Randox; SI1257) by following manufacturer’s instruction (Supplementary Table 1).

### Complete blood count analysis

Uncoagulated blood was collected in EDTA-coated tubes (BD Biosciences, USA) from overnight fasted animals and complete blood count was performed at the Health Science Animal Resource Centre, University of Calgary according to the approved protocol.

### Real-time RT PCR and immunoblotting

RNA was extracted from snap frozen tissues using Trizol reagent method (Invitrogen, USA) according to the manufacturer’s instructions^23^. Western blotting for cleaved caspase-3 and SCD-1 (from Cell Signaling used at 1:1000 dilution) was done using spleen and liver homogenates, respectively^59^. β-actin (Sigma) was used at 1:100,000 dilution. Uncropped scans of immunoblots are provided in the source data file. The PCR primer pairs is provided in Supplementary Table 2.

### Histopathology and Prussian iron staining

Colon from euthanized animals were excised and processed for presence of neutral mucins by periodic acid-Schiff’s (PAS) reagent using manufacturer’s instruuctions^23^. For total iron staining of spleen and liver tissues, Prussian iron blue staining was performed on formalin-fixed tissues using established protocol. Muc2 mucin was stained with anti-Muc2 antibody as previously described^60^.

### FISH and IgA^+^ bacteria quantification

FISH was performed to detect microbial penetrance through the small intestine and colonic mucosal surfaces^61^. In brief, Carnoy fixed tissues were embedded in paraffin and 5 μm thin sections were used to detect bacteria using the total bacteria probe EUB338 (50 ng/μl, final concentration, from Eqixon) incubated at 46 °C overnight. Nucleus was visualized using DAPI (Life Technologies). An Olympus FV1000 scanning confocal inverted microscope was used to visualize and record fluorescence in tissues. ImageJ software was used to calculate distance of bacteria from the epithelium^62^. IgA-coated bacteria in fecal samples from *Muc2*^−/−^ littermates were quantified and fecal samples from *Rag*^−/−^ animals were used as negative controls^29^.

### RBC isolation, PKH26 labeling, and reticulocytes staining

RBCs were isolated from uncoagulated blood of mice collected in ETDA tubes by repeated washing with HBSS (Invitrogen, USA) and labeled with PKH26 dye (Sigma, USA) using manufacturer’s protocol. Briefly, 2 × 10^7^ RBCs per ml were suspended in a conical polypropylene tube and centrifuged 1000 × *g* at 4 °C for 10 min and resuspended in 1 ml of 2x cell suspension diluent C. PKH26 dye was added to a final volume of 1 × 10^7^ cells/ml and 2 × 10^−6^ M PKH26. Cells and dye mixture was incubated for 10 min at room temperature in dark and staining was stopped by adding equal volume of fetal bovine serum incubated for 1 min. Labeled RBCs were washed twice with HBSS by centrifugation at 100 × *g* for 10 min and resuspended in 10^8^ RBCs per ml. For hemolysis-free serum collection, blood was collected in BD microtainer tubes without anticoagulant and stored at −80 °C for further analysis. Reticulocytes staining were performed from uncoagulated blood using modified Wrigh–Geimsa staining kit (Fisher Scientific) using standard protocol.

### In vitro bacterial growth analysis

Hemolysis-free serum samples (mice and patients) were heat inactivated at 56 °C for 30 min and used at 100 μl per well. Non-pathogenic bacteria (*E. coli* HB101, K-12) were used to determine bacterial growth in serum samples collected from DFO treated (2 h after DFO treatment) or untreated animal serum samples. Optical density was measured at 600 nm wavelength using a spectrophotometer. Hemolysis-free serum samples were used to determine bacterial growth. Gram-negative bacteria from tissue (MLNs and spleen) were homogenized in sterile PBS and grown selectively on MacConkey agar plate^63^.

### SCFA analysis

Cecal samples (150 mg) were added into 1000 μl of aqueous NaOH (0.005 M) containing D3 Caproic acid (internal standard). The samples were homogenized using lysing matrix for three cycles in a FastPrep-24 homogenizer (MP Biomedicals, Santa Anna, CA, USA). After homogenization, it was centrifuged at 14,000 × *g* for 20 min at 4 °C. The supernatant was derivatized under ultrasonication by adding 500 μl of propanol/pyridine mixture (3:2) and 300 μl of water. The derivatized SCFAs were extracted in 300 μl of hexane and analyzed using GC/MS^64^. SCFA in serum samples were analyzed at the Metabolomics Innovation Centre (TMIC), University of Alberta, AB, Canada.

### Hepatic lipid analysis via NMR

Snap frozen liver samples were used to analyze saturated, monounsaturated, and polyunsaturated fatty acids by ^1^H NMR spectroscopy^65^. In brief, liver samples (100 mg) were homogenized in a FastPrep-24 homogenizer (MP Biomedicals, CA) using 1.4 ceramic beads (Lysing Matrix D, MP Biomedicals) in 82% cold methanol. Following this, 66.6% chloroform was added to the homogenates and incubated for 10 min on ice. Samples were centrifuged at 14,000 × *g*, 10 min at 4 °C to separate aqueous and lipophilic layers which were dried separately in a Vacufuge concentrator 5301 (Eppendorf, Hamburg, Germany). Lipophilic extracts were then dissolved in deuterated chloroform containing octamethylcyclotetrasiloxane (OMS) as an internal standard. We used Bruker Advance 600 spectrometer (Bruker Biospin, Milton Canada) to acquire spectra using a standard pulse program (prnoesy 1d) operating at 600.22 MHz at 297 K (5 mm TXI Probe). Data were processed and profiled using AMIX version 3.9.14 (Bruker BioSpin). Lipid signals were integrated from the extracts relative to the OMS peak and then corrected for sample weight.

### Flow cytometry analysis

Single cell suspension was prepared from spleen, liver, and bone marrow and stained with fluorescently labeled antibodies using previously standardized protocol with the antibodies listed in Supplementary Table 3. Intracellular staining was performed following fixation and permeabilization using Fix/Perm transcription factor kit (BD biosciences). Live/dead cell and apoptotic cell discrimination done using 7-AAD and Annexin V staining (BD biosciences) and fixable viability dye 510. Data was acquired using BD FACS Canto machine and analyzed in FLOW JO software.

### Osmotic fragility test

Equal numbers of isolated RBCs from mice were added into increasing concentration of NaCl (0.0–0.9%, pH 7.4) buffered salt solution in final volume of 200 μl. These mixtures were incubated at 37 °C after gentle mixing for 30 min. Samples were centrifuged 2000 × *g* for10 min and supernatant was used to recorded O.D. @ 490 nm.

### Generation of bone marrow chimeric animals

A single dose of 9.50 Gy was given to *Muc2*^−/−^ littermates 6–8 weeks old and reconstituted with 5 × 10^5^ bone marrow cells per animal within 5 h by intravenous route. Animals were given 2 g/L neomycin sulfate ad libitum for 3 weeks and then intestinal microbiota was reconstituted by homologous fecal microbiota transfer (FMT) by oral gavage. FMT was done three times 3 days apart and experiments were carried out 10 days following the last FMT.

### Microscopy

16 h post RBC labeling (PKH26-PE, SIGMA) and i.v. transfer (1 × 10^9^ RBC in 300 μl/mouse) mice were injected i.v. with anti-mouse antibodies F4/80 coupled to Alexa647 (2 μg/mouse in 100 μl saline). 20 min post antibody application mice were necropsied and the whole spleen was placed in a cell culture dish covered with PBS and fixed underneath a glass coverslip to image random fields of view ex vivo. Intravital microscopy was performed using a Quorum WaveFx spinning disk confocal upright microscope (WaveFx, Quorum, Guelph, ON) with an Olympus BX51W1 body (Olympus, Center Valley, PA) equipped with a ×10/0.3 NA UPlan FLN objective and a Yokogawa CSU-10 scan head (Yokogawa Electric Corporation, Tokyo, Japan). Laser excitation at 561 and 640 nm (Cobalt, Stockholm, Sweden) was used and fluorescence was visualized with the appropriate emission band filters (593 ± 40 and 624 ± 40 nm, respectively, Semrock, Rochester, NY). Exposure time was constant at 400 ms (561 nm) and 200 ms (640 nm). Sensitivity settings were maintained at the same level for all experiments. A 512 × 512 pixel back-thinned EMCCD camera (model C9100-13, Hamamatsu, Bridgewater, NJ) was used for fluorescence detection. Volocity 6.1 acquisition software (Improvision Inc., Lexington, MA) was used to drive the confocal microscope. Images captured using the spinning disk were processed and analyzed in Volocity 4.20. Fluorescence-labeled F4/80-positive cells with or without positive RBC labeling in the spleen were enumerated by selecting five random fields of view at ×10 magnifications.

### Statistics

Data were analyzed using Graph Pad Prism version 5 software (Graph-Pad Software, San Diego, CA) and reported as the mean ± standard error of the mean (SEM). *t*-test was performed to ascertain differences between two groups. For three or more groups, ANOVA was used with a Bonferroni multiple comparison post-test. *p* values < 0.05 was considered significant.

### Reporting summary

Further information on research design is available in the Nature Research Reporting Summary linked to this article.

## Supplementary information

Supplementary Information

Reporting Summary

## Data Availability

The datasets generated and/or analyzed during the current study are available from the corresponding author on request. The source data for all figures are provided as a Source Data file.

## Source data

Source Data
