# Supplementary material for: Increased intestinal permeability exacerbates sepsis through reduced hepatic SCD-1 activity and dysregulated iron recycling
Source: Nat Commun. 2020 Jan 24;11:483. doi: 10.1038/s41467-019-14182-2 (PMC6981269; doi:10.1038/s41467-019-14182-2)
Supplement: Supplementary file 1 — Supplementary Information [file 41467_2019_14182_MOESM1_ESM.pdf]

Increased intestinal permeability exacerbates sepsis through reduced hepatic SCD-1 activity and dysregulated iron recycling

Kumar *et al*

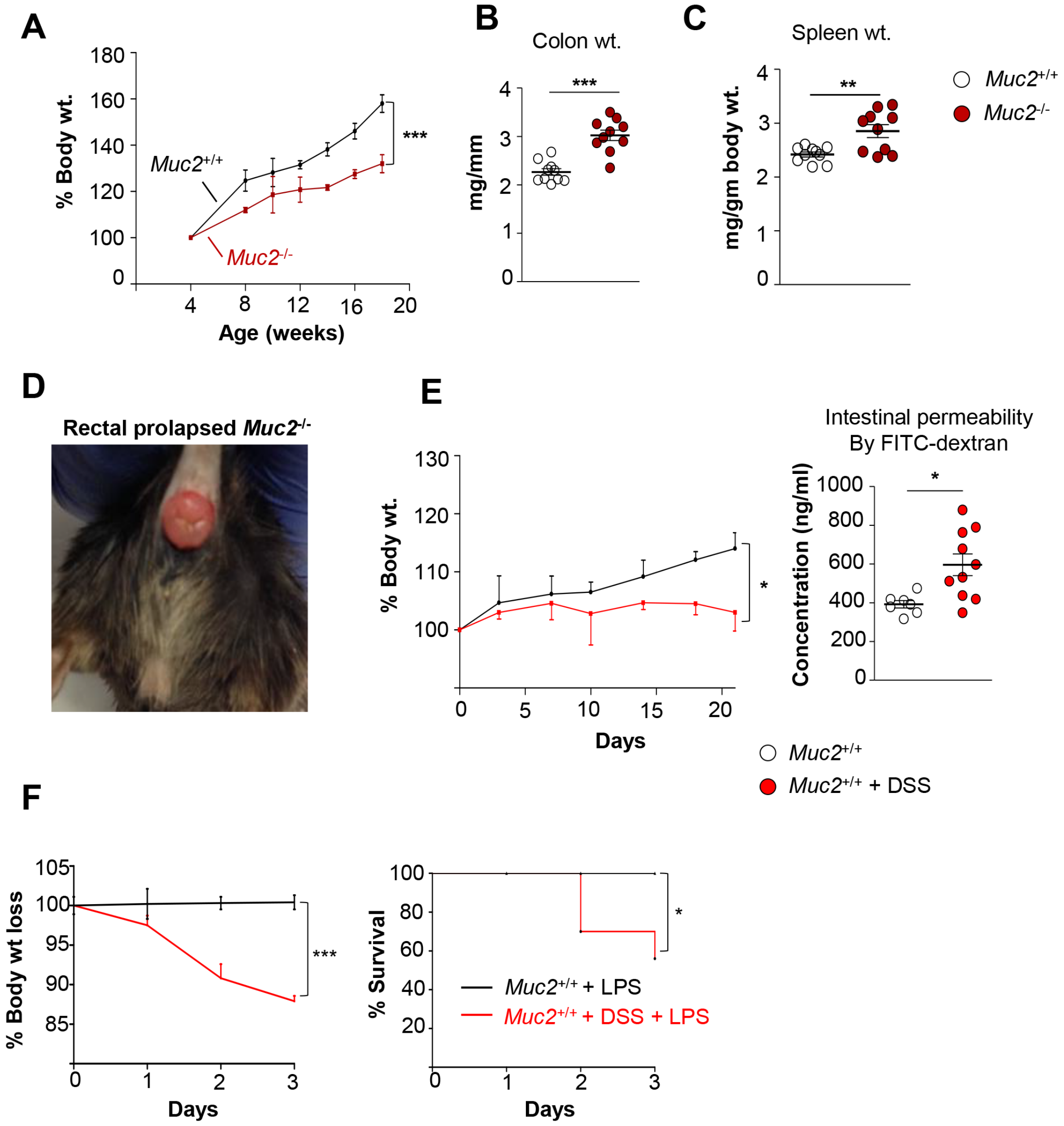
A
% Body wt.
Muc2+/+
Muc2-/-
***
Age (weeks)
B
Colon wt.
mg/mm
***
C
Spleen wt.
mg/gm body wt.
**
Muc2+/+
Muc2-/-
D
Rectal prolapsed Muc2-/-
E
% Body wt.
*
Days
Intestinal permeability
By FITC-dextran
Concentration (ng/ml)
*
Muc2+/+
Muc2+/+ + DSS
F
% Body wt loss
***
Days
% Survival
*
Muc2+/+ + LPS
Muc2+/+ + DSS + LPS
Days

Supplementary Figure 1: *Muc2$^{-/-}$* mice exhibit higher inflammation basally. A. Basal body weight gain comparison shows significantly less weight gain by *Muc2$^{-/-}$* as compared to *Muc2$^{+/+}$* littermates. (n=10). **B-C**. Colon and spleen weight in *Muc2$^{-/-}$* were significantly higher than *Muc2$^{+/+}$* littermates as a result of ongoing low-grade inflammation. **B**. Colon was excised, flushed with PBS to remove fecal matter, weighed, and normalized with its length to calculate weight. **C**. Spleen was surgically excised and weight and normalized with the body weight of animal. (n=6). **D**. Representative rectal prolapsed image of *Muc2$^{-/-}$* mice. **E-F**. Colitis was induced in *Muc2$^{+/+}$* littermates using continuous low dose DSS (0.75%) for three weeks and at the end of DSS treatment, sensitivity to sepsis was evaluated by challenging animals with LPS at 5mg/kg body weight intraperitoneally (n=6-10). **E**. *Muc2$^{+/+}$* littermates treated with low dose DSS gained significantly less body weight as compared to age matched untreated controls. DSS treated mice exhibited significantly increased intestinal permeability as determined by FITC-dextran at the end of treatment. **F**. LPS administration caused higher body weight loss and mortality in DSS treated *Muc2$^{+/+}$* as compared to untreated controls. Representative data from 2 independent experiments; paired one-way ANOVA. *$p < 0.05$, **$p < 0.01$ and ***$p < 0.001$.

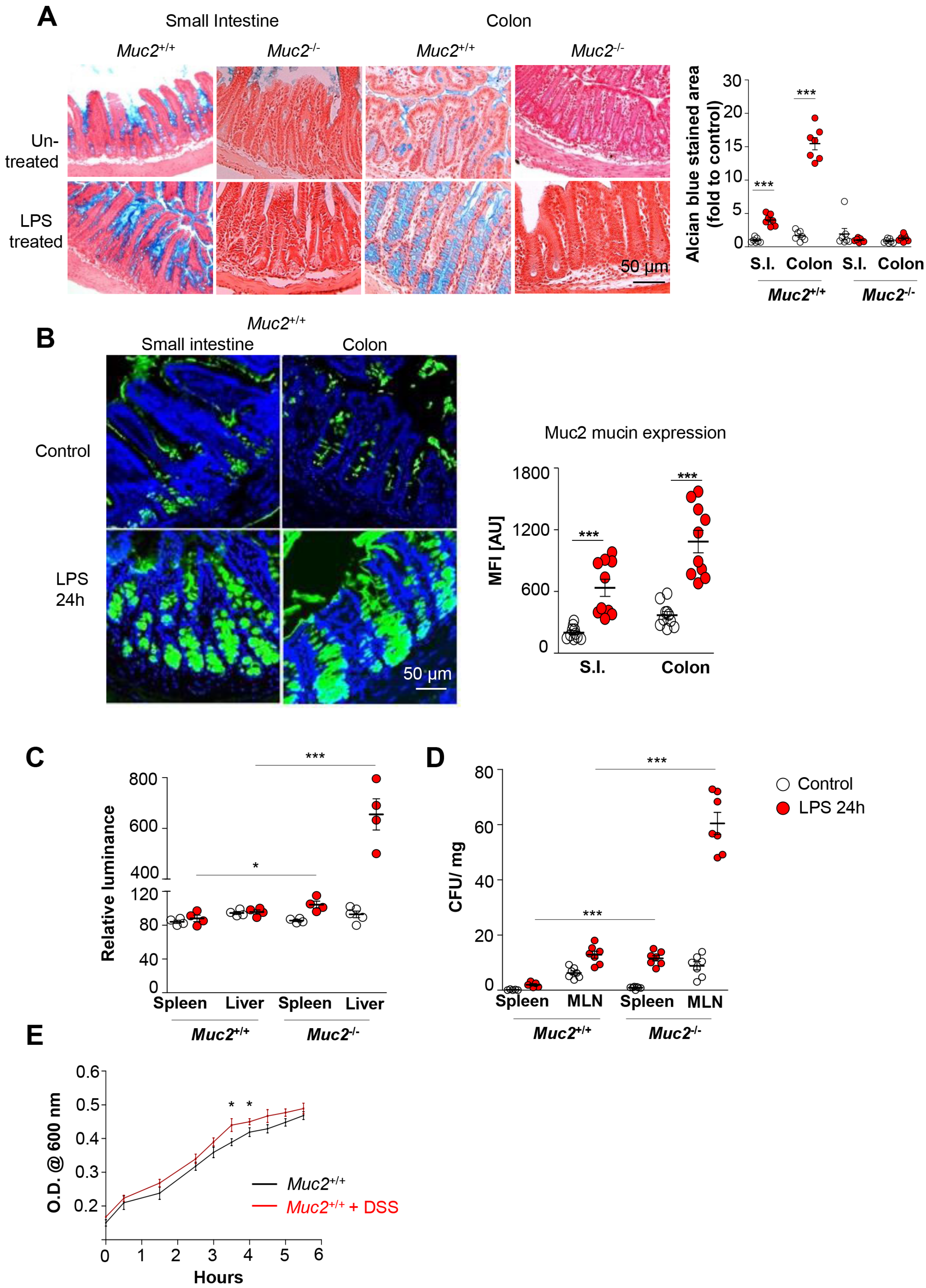
A
Small Intestine
Colon
Muc2+/+
Muc2-/-
Muc2+/+
Muc2-/-
Un-
treated
LPS
treated
50 µm
Alcian blue stained area
(fold to control)
30
20
15
10
5
0
***
***
S.I.
Colon
S.I.
Colon
Muc2+/+
Muc2-/-
B
Muc2+/+
Small intestine
Colon
Control
LPS
24h
50 µm
Muc2 mucin expression
MFI [AU]
1800
1200
600
0
***
***
S.I.
Colon
C
Relative luminance
800
600
400
120
80
40
0
***
*
Spleen
Liver
Spleen
Liver
Muc2+/+
Muc2-/-
D
CFU/ mg
80
60
40
20
0
***
***
Spleen
MLN
Spleen
MLN
Muc2+/+
Muc2-/-
Control
LPS 24h
E
O.D. @ 600 nm
0.6
0.5
0.4
0.3
0.2
* *
Muc2+/+
Muc2+/+ + DSS
0
1
2
3
4
5
6
Hours

Supplementary Figure 2. Muc2 mucin protects against sepsis. **A**. *Muc2*$^{-/-}$ littermates were challenged with LPS and monitored for 3 days. Colonic tissue was excised at the end of experiment and immediately fixed in Carnoy's to preserve the mucus layer. Fixed tissues were embedded in paraffin and sectioned at 5μm thickness. Tissue sections were stained with Alcian blue to detect acidic mucin and counterstained with nuclear fast for nuclei and cytoplasm. Representative images are from 4 independent experiment, n=5. Bar graph shows cumulative data from a single experiment, quantification of Alcian blue stained area and plotted as fold change after normalization with respective control. **B**. Robust secretion of Muc2 mucus in the small intestine (SI) and colon of *Muc2*$^{+/+}$ littermates following LPS administration. Muc2 mucin was stained with anti-Muc2 antibody (green) and nucleus with DAPI (blue). Representative confocal images are from three independent experiments (n=4-7). Graph shows quantification of Muc2 mucin using imageJ and plotted as Means ± SEM. **C**. Luminescence of *XEN*-14 *E. coli* was recorded in the spleen and liver from LPS treated animals after 24h. Organs were homogenized in sterile PBS and luminescence was recorded using FB12 tube Luminometer (Berthold, Germany) (n=4). Graph shows cumulative data from two independent experiments. **D**. CFU of translocated intestinal bacteria calculated in spleen and mesenteric lymph nodes of *Muc2*$^{-/-}$ littermates 24h post LPS challenged. Tissue was homogenized in sterile PBS and plated on MacConkey agar plates overnight at 37° C. CFU was normalized with the dilution factor and plotted as means ± SEM (n=4-6). **E**. Higher bacterial growth in heat inactivated hemolysis free serum of DSS treated *Muc2*$^{+/+}$ animals (n=6-10). Representative of two independent experiments. *$p < 0.05$, and ***$p < 0.001$.

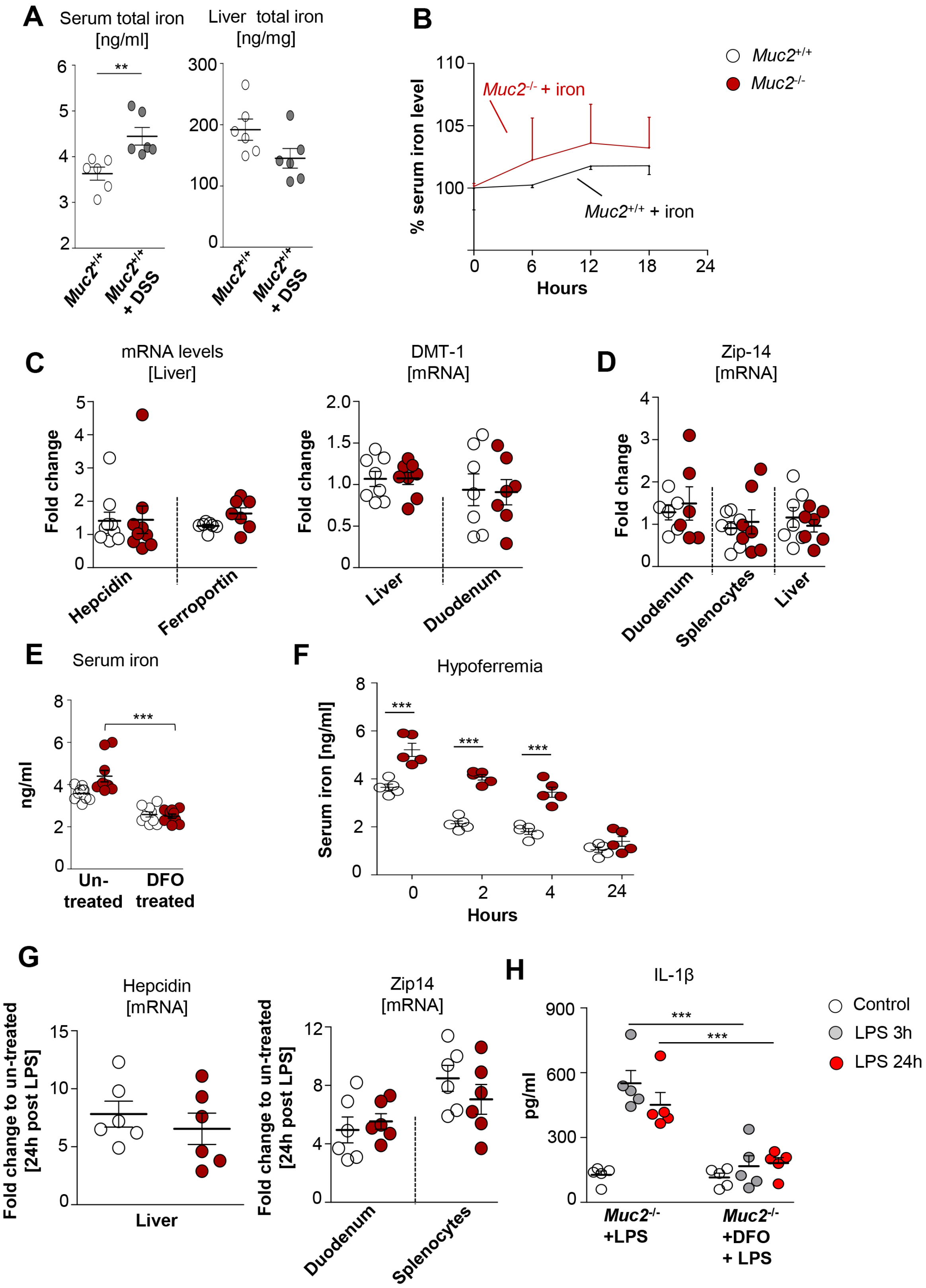
A
Serum total iron [ng/ml]
Liver total iron [ng/mg]
**
Muc2+/+
Muc2+/+ + DSS
B
% serum iron level
Muc2-/- + iron
Muc2+/+ + iron
Hours
Muc2+/+
Muc2-/-
C
mRNA levels [Liver]
Fold change
Hepcidin
Ferroportin
DMT-1 [mRNA]
Liver
Duodenum
D
Zip-14 [mRNA]
Duodenum
Splenocytes
Liver
E
Serum iron
ng/ml
***
Un-treated
DFO treated
F
Hypoferremia
Serum iron [ng/ml]
***
Hours
G
Hepcidin [mRNA]
Fold change to un-treated [24h post LPS]
Liver
Zip14 [mRNA]
Duodenum
Splenocytes
H
IL-1β
pg/ml
***
Muc2-/- +LPS
Muc2-/- +DFO + LPS
Control
LPS 3h
LPS 24h

Supplementary Figure 3. ***Muc2***$^{-/-}$ **mice exhibit higher circulatory iron load basally. A**. Higher circulatory total iron levels were observed in hemolysis free serum of *Muc2*$^{+/+}$ littermates when colitis was induced with 0.75% DSS for three weeks (n=8). Data represents two independent experiments; Means ± SEM, paired Student's t test. **B**. Serum total iron levels in *Muc2*$^{-/-}$ littermates after gavaging 2mg/kg body weight of an aqueous solution of ferrous sulfate in tap water. Results suggest no significant difference in dietary iron absorption between the two genotypes, n=4-5 paired one-way ANOVA. **C**. Real time RT-PCR showing comparable transcript levels of key iron regulatory genes hepcidin, ferroportin and DMT-1 expressed in the liver and ZIP-14 expressed in the duodenum of *Muc2*$^{-/-}$ and *Muc2*$^{+/+}$ littermates (n=7-8). Data represents three independent experiments; Means ± SEM, paired Student's t test. **D**. Comparable transcript expression levels of Zip14 gene in duodenum, splenocytes and liver of *Muc2*$^{-/-}$ and *Muc2*$^{+/+}$ littermates as analysed by real time RT-PCR (n=6-7). **E**. Attenuated serum total iron levels in DFO treated *Muc2*$^{+/+}$ and *Muc2*$^{-/-}$ littermates (n=4-6). Representative data are from two independent experiments. **F**. Delayed hypoferremic response in *Muc2*$^{-/-}$ following LPS challenge (n=5). Representative data of three experiments paired one-way ANOVA. **G**. Real time RT-PCR showing transcript levels of liver hepcidin and Zip14 in duodenum and spleen tissue collected 24h post LPS treatment from *Muc2*$^{+/+}$ and *Muc2*$^{-/-}$ mice (n=6). **H**. Attenuation of pro-inflammatory cytokine IL-1β levels in the serum of DFO pre-treated *Muc2*$^{-/-}$ animals following LPS challenge reiterating a role for excess of iron in susceptibility towards sepsis (n=5). Representative data from three independent experiments; paired one-way ANOVA. **$p < 0.01$ and ***$p < 0.001$.

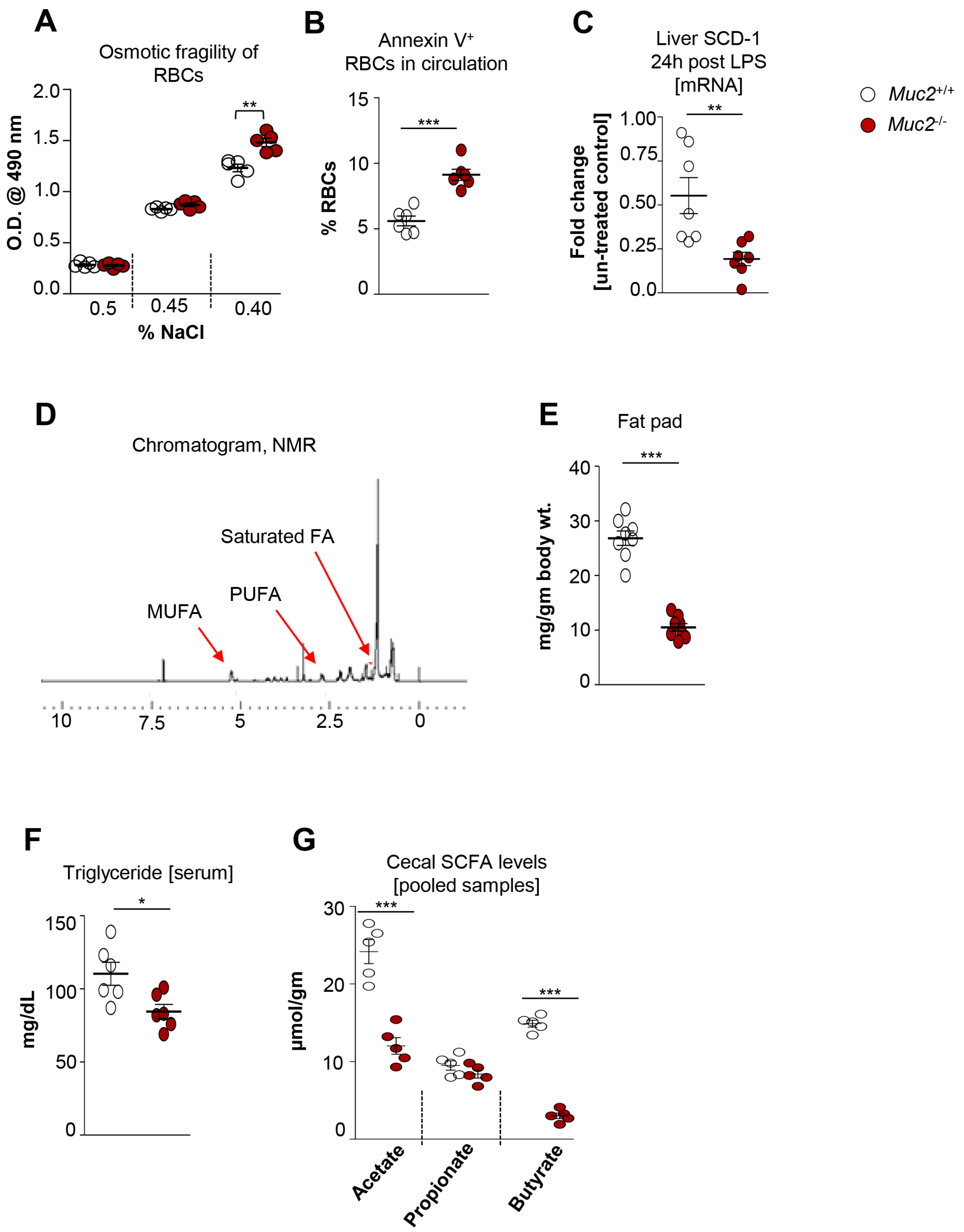
A
Osmotic fragility of RBCs
O.D. @ 490 nm
2.0
1.5
1.0
0.5
0.0
**
0.5
0.45
0.40
% NaCl
B
Annexin V+ RBCs in circulation
% RBCs
15
10
5
0
***
C
Liver SCD-1 24h post LPS [mRNA]
Fold change [un-treated control]
1.00
0.75
0.50
0.25
0.0
**
Muc2+/+
Muc2-/-
D
Chromatogram, NMR
Saturated FA
PUFA
MUFA
10
7.5
5
2.5
0
E
Fat pad
mg/gm body wt.
40
30
20
10
0
***
F
Triglyceride [serum]
mg/dL
150
100
50
0
*
G
Cecal SCFA levels [pooled samples]
µmol/gm
30
20
10
0
***
***
Acetate
Propionate
Butyrate

Supplementary Figure 4. Higher RBC fragility in *Muc2*$^{-/-}$ mice. A. Higher osmotic fragility levels of *Muc2*$^{-/-}$ RBCs when subjected to varying concentration of NaCl (n=5). Representative data are from three independent experiments paired one-way ANOVA. **B**. Increased population of apoptotic RBCs at basal levels in the circulation of *Muc2*$^{-/-}$ animals. n=5, paired Student's t test. **C**. SCD-1 transcript levels in liver samples in *Muc2*$^{+/+}$ and *Muc2*$^{-/-}$ littermates following 24h post LPS treatment (n=7). Representative data are from three independent experiments. **D**. Representative chromatogram of NMR analysis showing MUFA, PUFA and saturated fat peaks in liver samples. **E**. Depleted fat pad deposits in *Muc2*$^{-/-}$ as compared to *Muc2*$^{+/+}$ littermates suggest ongoing systemic inflammation. Inguinal fat pad was collected only from the right hand side and its weight was normalized to the body weight of animals. Means ± SEM, n=8-10, paired Student's t test. **F**. Lowered serum triglyceride content manifests lesser hepatic fatty acid synthesis activity in *Muc2*$^{-/-}$ animals. Means ± SEM, n=8-10, paired Student's t test. **G**. SCFA analysis quantified by GC-MS in cecal contents of *Muc2*$^{-/-}$ littermates shows significantly attenuated production of the major SCFAs acetate, propionate and butyrate (n=6). Representative data from two independent experiments. $^{*}p < 0.05$, $^{**}p < 0.01$ and $^{***}p < 0.001$.

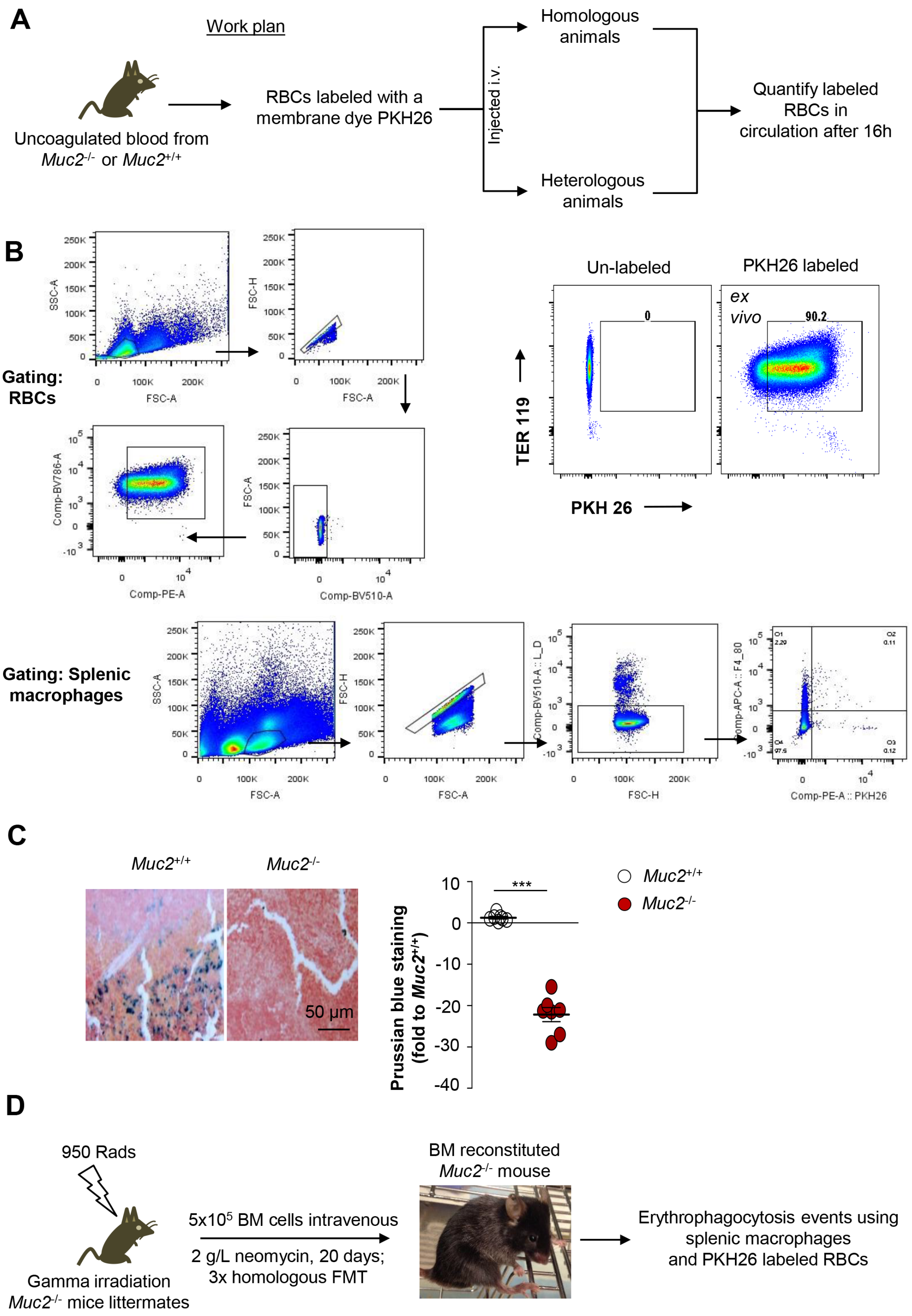
A
Work plan
Uncoagulated blood from
Muc2-/- or Muc2+/+
RBCs labeled with a
membrane dye PKH26
Injected i.v.
Homologous
animals
Heterologous
animals
Quantify labeled
RBCs in
circulation after 16h
B
Gating:
RBCs
Un-labeled
PKH26 labeled
ex
vivo
0
90.2
TER 119
PKH 26
Gating: Splenic
macrophages
C
Muc2+/+
Muc2-/-
50 µm
Prussian blue staining
(fold to Muc2+/+)
***
D
950 Rads
Gamma irradiation
Muc2-/- mice littermates
5x10^5 BM cells intravenous
2 g/L neomycin, 20 days;
3x homologous FMT
BM reconstituted
Muc2-/- mouse
Erythrophagocytosis events using
splenic macrophages
and PKH26 labeled RBCs

Supplementary Figure 5. Inefficient erythrophagocytosis by *Muc2*$^{-/-}$ macrophages. A. Work plan schematics. Uncoagulated blood from *Muc2*$^{-/-}$ and *Muc2*$^{+/+}$ littermates was collected in EDTA coated tubes and RBCs was isolated and labelled with a lipophilic dye PKH26 *ex vivo*. Labelled RBCs were then injected back intravenously via tail vein into homologous/heterologous littermates to determine their clearance at different time points. **B**. Gating strategy used to analyse PKH26 labelled RBCs and erythrophagocytosis by splenic macrophages. Representative dot plots (one of three experiments) showing isolation and *ex vivo* RBCs labelling efficiency of PKH26 dye and anti-TER119 antibody. **C**. Spleen samples from saline perfused euthanized animals were collected, formalin fixed, paraffin embedded, sectioned, and stained with Prussian blue stain for iron. Representative Prussian blue stained spleen samples shows lower iron deposits at basal level in *Muc2*$^{-/-}$ littermates. Representative image from four independent experiments. Cumulative quantitative analysis of four experiments. **D**. Generation of bone marrow chimeras using *Muc2*$^{-/-}$ littermates. Animals were exposed to a single lethal dose of 950 Rads and reconstituted with donor 5 x 10^5 BM cells within 5h intravenously and provided with 2gm/L neomycin water. Means ± SEM, paired Student's t test. ***$p < 0.001$.

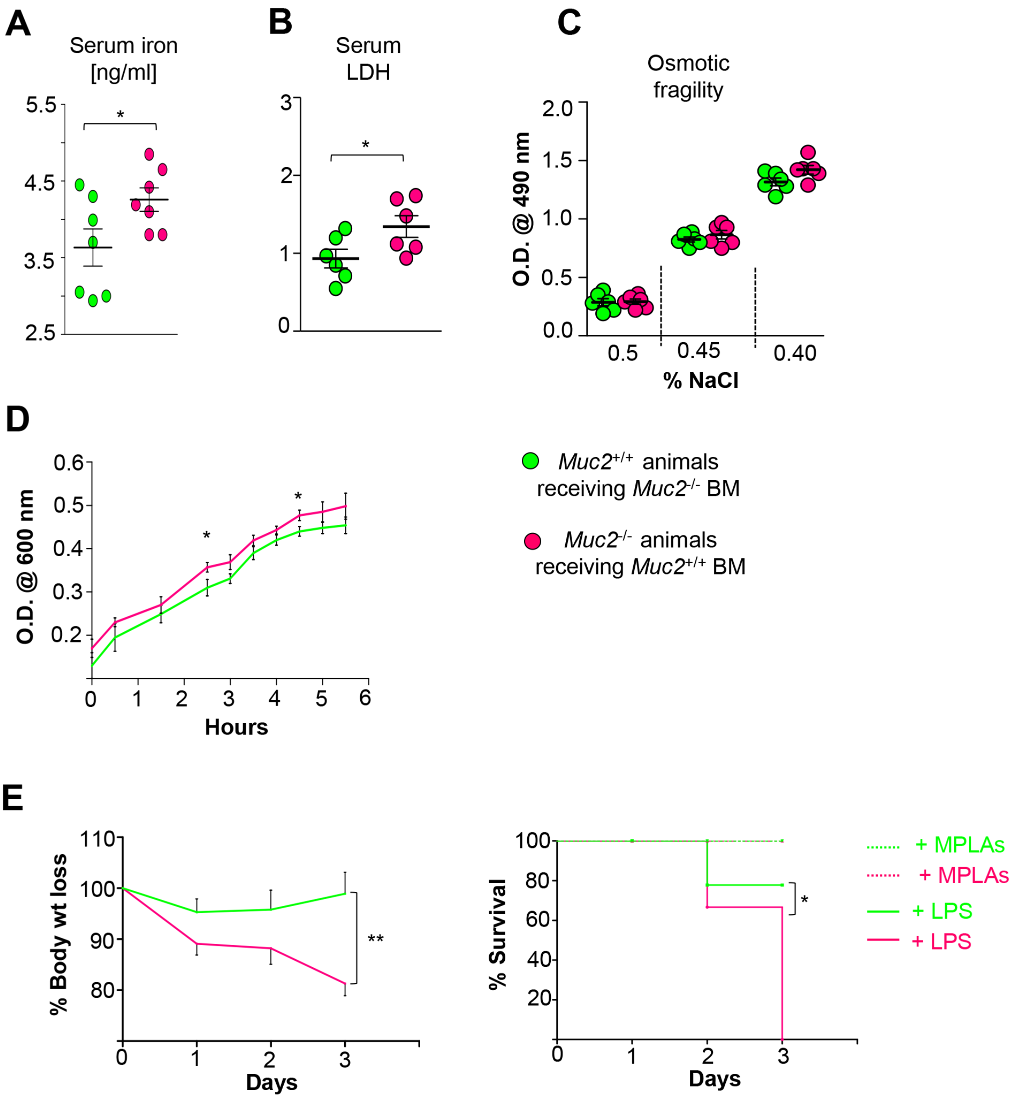
A
Serum iron [ng/ml]
B
Serum LDH
C
Osmotic fragility
O.D. @ 490 nm
% NaCl
D
O.D. @ 600 nm
Hours
Muc2+/+ animals receiving Muc2-/- BM
Muc2-/- animals receiving Muc2+/+ BM
E
% Body wt loss
Days
% Survival
+ MPLAs
+ MPLAs
+ LPS
+ LPS

Supplementary Figure 6. Reduced erythrophagocytosis by *Muc2*$^{-/-}$ macrophages. A. Reduced levels of total serum iron levels in *Muc2*$^{-/-}$ mice receiving *Muc2*$^{+/+}$ bone marrow as compared to control (n=4-8). Representative data are from two independent experiments. **B**. High serum LDH levels in *Muc2*$^{-/-}$ littermates receiving *Muc2*$^{+/+}$ bone marrow (n=6). **C**. Comparable levels of osmotic fragility index of RBCs isolated from bone marrow chimeras (n=6). **D**. Higher bacterial growth in serum of *Muc2*$^{-/-}$ receiving *Muc2*$^{+/+}$ bone marrow as compared to *Muc2*$^{+/+}$ receiving *Muc2*$^{-/-}$ bone marrow (n=4-8). **E**. Significantly high body weight loss and increased mortality among *Muc2*$^{-/-}$ receiving *Muc2*$^{+/+}$ bone marrow after LPS challenge as compared to *Muc2*$^{+/+}$ littermates receiving *Muc2*$^{-/-}$ bone marrow (n=8). Representative data from two independent experiments. *$p < 0.05$, and **$p < 0.01$.

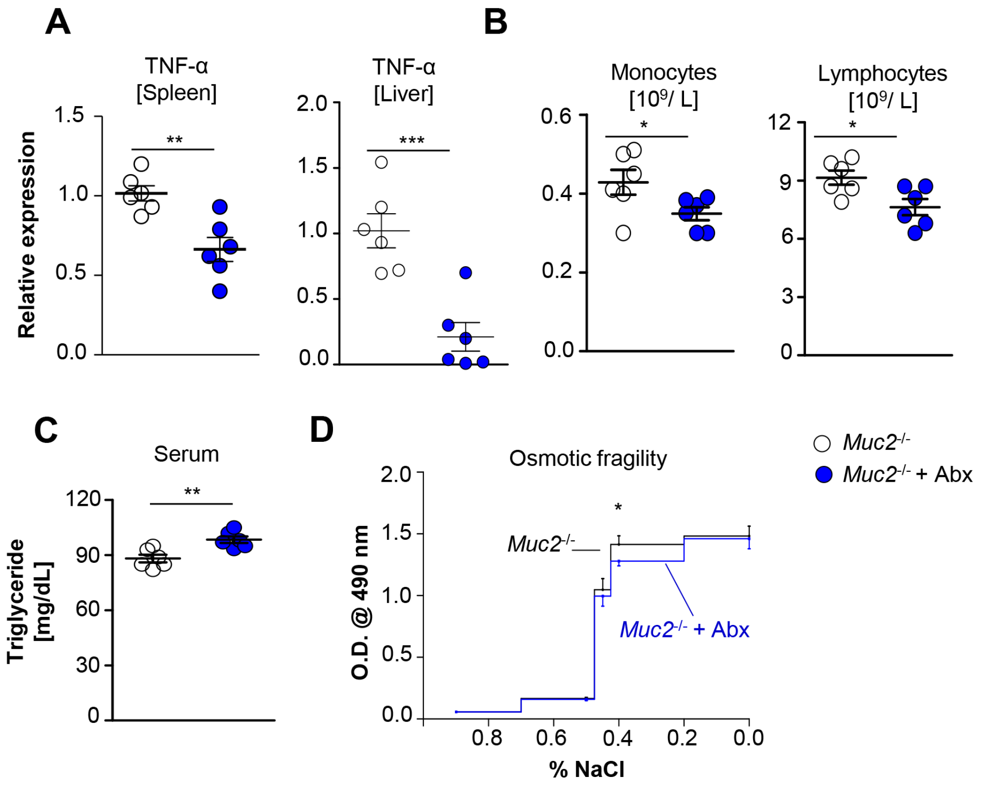

A
TNF-α
[Spleen]
Relative expression
1.5
1.0
0.5
0.0
**
TNF-α
[Liver]
2.0
1.5
1.0
0.5
0.0
***
B
Monocytes
[10^9/ L]
0.6
0.4
0.2
0.0
*
Lymphocytes
[10^9/ L]
12
9
6
3
0
*
C
Serum
Triglyceride
[mg/dL]
120
90
60
30
0
**
D
Osmotic fragility
O.D. @ 490 nm
2.0
1.5
1.0
0.5
0.0
*
Muc2-/-
Muc2-/- + Abx
0.8
0.6
0.4
0.2
0.0
% NaCl
Muc2-/-
Muc2-/- + Abx

Supplementary Figure 7. Decreases hepatic lipogenesis in *Muc2*$^{-/-}$ mice. *Muc2*$^{-/-}$ animals were treated with a cocktail of broad-spectrum Abx to reduce gut microbial load and bacterial penetrance/translocation into the tissues. **A**. Real time RT-PCR showing a reduction in TNF-α mRNA levels in the spleen and liver following Abx treatment (n=6). Data are representative of four independent experiments. **B**. CBC analysis confirmed lowered monocytes and lymphocytes counts in the circulation of *Muc2*$^{-/-}$ animals following Abx treatment (n=4-8). **C**. Abx treatment increased serum triglyceride content and restored hepatic fatty acid synthesis activity in *Muc2*$^{-/-}$ animals (n=6). **D**. Reduction in osmotic fragility of RBCs isolated from Abx treated *Muc2*$^{-/-}$ animals to varying concentration of NaCl (n=6). Representative data from two independent experiments. *$p < 0.05$, **$p < 0.01$ and ***$p < 0.001$.

Supplementary Table 1: List of chemicals (Catalogue number)

Lipopolysaccharide	Sigma; L3024
Iron quantification kit	Sigma; MAK025
Deferoxamine	Sigma; D9533
MPLAs TLR4 ligand	InvivoGen; 1246298-63-4
LDH assay kit	Abcam; ab102526
XEN14 E.coli	Perkin Elmer; 119223
SCD-1 antibody	Cell Signalling; 2438
cleaved caspase-3 antibody	Cell Signalling; 9664
Potassium ferrocyanide trihydrate	Sigma; P3289
Neomycin Sulfate	EMD Millipore; 4801-25GM
PKH26 dye	Sigma; PKH26GL
Alcian blue, pH 2.5 stain kit	Diagnostic Biosystems; KT003
Dextran Sulfate Sodium Salt (DSS)	Alfa Aesar; J63606
TIBC kit	Randox; TI1010
Serum iron quantification kit	Randox; SI257
Mouse Ferritin ELISA kit	Abcam; ab1557713
Lipocalin 2 (NGAL) antibody	Santa Cruz; sc515876

Supplementary Table 2: List of oligonucleotides

SCD-1; Forward	5'CCGGAGACCCTTAGATCGA3'
SCD-1; Reverse	5'TAGCCTGTAAAAGATTTCTGCAAACC3'
TNFα; Forward	5'CACCACGCTCTTCTGTCTAC3'
TNFα; Reverse	5'AGAAGATGATCTGAGTGTGAGG3'
IL-1β; Forward	5'GAAGAAGAGCCCATCCTCTG3'
IL-1β; Reverse	5'TCATCTCGGAGCCTGTAGTG3'
Hepcidin; Forward	5'TTGCGATACCAATGCAGAAG3'
Hepcidin; Reverse	5'TGCAACAGATACCACACTGG3'
Ferroportin; Forward	5'TGGATGGGTCCTTACTGTCTGCTAC3'
Ferroportin; Reverse	5'TGCTAATCTGCTCCTGTTTTCTCC3'
DMT-1; Forward	5'GGCTTTCTTATGAGCATTGCCTA3'
DMT-1; Reverse	5'GGAGCACCCAGAGCAGCTTA3'
β-actin: Forward	5'TGGGGTGTTGAAGGTCTC3'
β-actin: Reverse	5'CTACAATGAGCTGCGTGTG3'
ZIP14 A- Forward	TTCCTCAGTGTCTCACTGATTAA
ZIP14 A- Reverse	GGAAAAGGGCGTTAGAGAGC
Pparα- Forward	CTGCAGAGCAACCATCCAGAT
Pparα- Reverse	GCCGAAGGTCCACCATTTT
Cpt1α- Forward	TGGCATCATCACTGGTGTGTT
Cpt1α - Reverse	GTCTAGGGTCCGATTGATCTTTG
Acox1- Forward	GCCCAACTGTGACTTCCATC
Acox1- Reverse	GCCAGGACTATCGCATGATT
SLC5A8- Forward	TCGAGTTGGCGAAGGGGACCA
SLC5A8- Reverse	ATGCCTTGGCGGCAGTCACC
GPR109A - Forward	ATGGCGAGGCATATCTGTGTAGCA

GPR109A - Reverse	TCCTGCCTGAGCAGAACAAGATGA
FAS- Forward	TTCCAAGACGAAAATGATGC
FAS- Reverse	AATTGTGGGATCAGGAGAGC
SREBP1- Forward	CGGAAGCTGTCGGGGTAG
SREBP1- Reverse	GTTGTTGATGAGCTGGAGCA

Supplementary Table 3: List of antibodies for Flow cytometry

TER119- BV786	BD Pharmingen; 740875
F4/80- AF647	BD Pharmingen; 565854
CD11b- PerCPCy5.5	BD Pharmingen; 561114
Ly6C- PE-Cy7	BD Pharmingen; 560593
CD45; V450	BD Pharmingen; 560501
Ly6G- FITC	BD Pharmingen; 127605
CD3-FITC	BD Pharmingen; 561798
CD19-FITC	BD Pharmingen- 561740
Mouse Th17/Treg phenotyping kit	BD Pharmingen; 560767
PE Annexin V Apoptosis detection kit	BD Pharmingen; 5306537
